# A developmental role for the chromatin-regulating CoREST complex in the cnidarian *Nematostella vectensis*

**DOI:** 10.1186/s12915-022-01385-1

**Published:** 2022-08-23

**Authors:** James M. Gahan, Lucas Leclère, Maria Hernandez-Valladares, Fabian Rentzsch

**Affiliations:** 1 Sars International Centre for Marine Molecular Biology, University of Bergen, Thormøhlensgate 55, 5006 Bergen, Norway; 2 Sorbonne Université, CNRS, Laboratoire de Biologie du Développement de Villefranche-Sur-Mer (LBDV), 06230 Villefranche-sur-Mer, France; 3 Department of Physical Chemistry, University of Granada, Campus Fuentenueva s/n, 18071 Granada, Spain; 4 Proteomics Facility of the University of Bergen (PROBE), University of Bergen, 5020 Bergen, Norway; 5 Department for Biological Sciences, University of Bergen, Thormøhlensgate 53, 5006 Bergen, Norway

**Keywords:** Evolution, Chromatin modification, Gene regulation, Development, CoREST, Lsd1, KDM1A, Cnidaria

## Abstract

**Background:**

Chromatin-modifying proteins are key players in the regulation of development and cell differentiation in animals. Most chromatin modifiers, however, predate the evolution of animal multicellularity, and how they gained new functions and became integrated into the regulatory networks underlying development is unclear. One way this may occur is the evolution of new scaffolding proteins that integrate multiple chromatin regulators into larger complexes that facilitate coordinated deposition or removal of different chromatin modifications. We test this hypothesis by analyzing the evolution of the CoREST-Lsd1-HDAC complex.

**Results:**

Using phylogenetic analyses, we show that a bona fide CoREST homolog is found only in choanoflagellates and animals. We then use the sea anemone *Nematostella vectensis* as a model for early branching metazoans and identify a conserved CoREST complex by immunoprecipitation and mass spectrometry of an endogenously tagged Lsd1 allele. In addition to CoREST, Lsd1 and HDAC1/2 this complex contains homologs of HMG20A/B and PHF21A, two subunits that have previously only been identified in mammalian CoREST complexes. NvCoREST expression overlaps fully with that of NvLsd1 throughout development, with higher levels in differentiated neural cells. *NvCoREST* mutants, generated using CRISPR-Cas9, fail to develop beyond the primary polyp stage, thereby revealing essential roles during development and for the differentiation of cnidocytes that phenocopy *NvLsd1* mutants. We also show that this requirement is cell autonomous using a cell-type-specific rescue approach.

**Conclusions:**

The identification of a *Nematostella* CoREST-Lsd1-HDAC1/2 complex, its similarity in composition with the vertebrate complex, and the near-identical expression patterns and mutant phenotypes of NvCoREST and NvLsd1 suggest that the complex was present before the last common cnidarian-bilaterian ancestor and thus represents an ancient component of the animal developmental toolkit.

**Supplementary Information:**

The online version contains supplementary material available at 10.1186/s12915-022-01385-1.

## Background

Understanding the evolution of animal development and cell differentiation requires analysis of the gene regulatory programs that direct these processes. In recent years, comparisons between animal groups have found a remarkable degree of conservation of transcription factors and signaling pathways throughout the animal kingdom [1–6]. This suggests that changes in the repertoire of these genes alone cannot explain the diversification of developmental processes. Regulation of chromatin has been shown to be another essential aspect of transcription during development [7–9], but its potential role in the evolution of developmental gene regulation has received little attention.

Most chromatin regulators are ancient and predate the evolution of animal multicellularity [10]. One possible way to evolve new roles in developmental programs is the integration of chromatin regulators into multiprotein complexes that facilitate the coordination of different regulatory (e.g. enzymatic) activities and/or facilitate targeting to specific genomic loci. Here, we use the CoREST complex to explore this scenario in an early-diverging group of animals.

The CoREST complex was initially discovered in mammals as a complex required for repression of neuronal genes. It consists of three core proteins: Lysine-specific demethylase 1 (Lsd1/Kdm1a), Co-repressor of REST (CoREST), and Histone deacetylase 1/2 (HDAC1/2), as well as several other subunits [11–18]. The CoREST complex is capable of repressing transcription through coordinated deacetylation and demethylation of histones [19–23]. The Lsd1-CoREST interaction and the tertiary complex have been extensively examined, both structurally and biochemically, and CoREST has been shown to be required for demethylation of lysine 4 of histone H3 (H3K4) on nucleosome substrates by Lsd1 in mammals [20, 22, 24–28]. The presence of this complex has been shown in mammals [15], *Drosophila melanogaster* [29], and *Caenorhabditis elegans* [30, 31] indicating it is at least conserved throughout Bilateria. The CoREST complex and CoREST proteins have been shown to play roles in the differentiation and homeostasis of various tissues, including the nervous system [32–37], epidermis [38], immune system [39], and the hematopoietic system [40] in mammals. In *Drosophila,* dLsd1 and CoRest play roles in spermatogenesis and follicle cell differentiation in the ovary [29, 41, 42] while in *C. elegans* homologs of both are involved in reprogramming of the zygotic genome after fertilization, something which is conserved in mice [43–45].

*Nematostella vectensis*, the starlet sea anemone, represents a particularly attractive system to investigate the evolution of animal development. It is a member of the phylum Cnidaria, the sister group to the bilaterian animals, and therefore possesses an informative phylogenetic position which, through comparative analysis, can unveil aspects of early animal evolution. In addition, a diverse array of experimental tools and resources is available for *Nematostella* [46, 47]. Like most cnidarians, *Nematostella* has a simple adult body plan, known as a polyp, which resembles a tube with an opening at one end (oral) which serves as the mouth/anus and is surrounded by a ring of tentacles. Adults are dioecious and release sperm/eggs into the water column where fertilization occurs. Development proceeds through a hollow blastula which gastrulates via invagination to generate the two germ layers: ectoderm and endoderm. Following gastrulation, the animal develops into a free-swimming planula larva which eventually settles and metamorphoses into a primary polyp which will feed and grow to sexual maturity in approximately three months [48–50].

We have previously shown that the *Nematostella* ortholog of *Lsd1*, *NvLsd1*, in expressed ubiquitously throughout development but that its levels are developmentally regulated and are specifically high in differentiated neural cells relative to their progenitors. Using a mutant allele, we have also shown that loss of *NvLsd1* leads to a range of developmental abnormalities, the most pronounced of which is the almost total loss of differentiated cnidocytes, cnidarian-specific neural cells [51]. Here, by interrogating the interactome of NvLsd1 we found that the CoREST complex is conserved in *Nematostella*. We show that NvLsd1 and NvCoREST are expressed in precisely the same fashion throughout development. Using two mutant lines we show that loss of *NvCoREST* phenocopies loss of *NvLsd1* and that the CoREST complex is required for normal development and cnidocyte differentiation in *Nematostella*.

## Results

### The CoREST complex is present in *Nematostella vectensis*

**Fig. 1:**
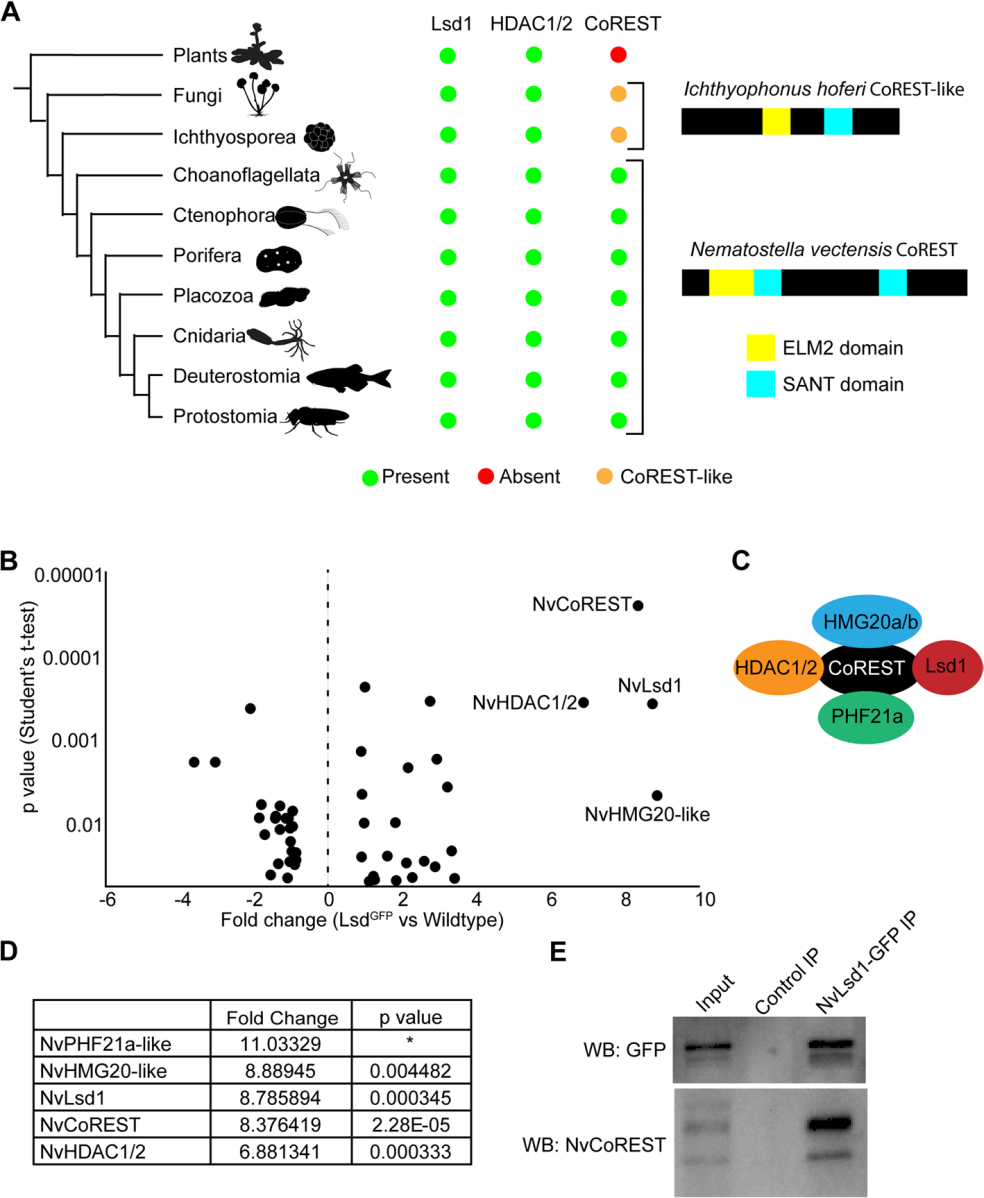
The CoREST complex is present in *Nematostella*. **A** Phylogenetic tree showing the presence/absence of Lsd1, HDAC1/2, and CoREST orthologs in the different groups. The tree on the left shows the relationships between the different clades analyzed. Green circles indicate the presence of orthologs within that group while red circles indicate their absence. A yellow circle indicates the groups where we see a CoREST-like protein containing only a single SANT domain. On the right are two representative protein sequences from *Nematostella vectencis* and *Ichthyophonus hoferi*. **B** Dot plot showing proteins derived from a comparison of Co-IP with GFP-Trap beads from *NvLsd1*^*GFP*^ and wild-type planula. Data is derived from three independent biological replicates. Only proteins with *p*-value < 0.05 (Student’s *t*-test, two-tailed) are shown. Fold change (FC) in the *NvLsd*^*GFP*^ sample over wildtype is shown on the *x*-axis and *p*-value on the *y*-axis. The four proteins with the highest FC are annotated with names. **C** Schematic representation of the human CoREST complex as first identified [15] **D** Table showing the fold change and *p*-value for the five most enriched proteins in the *NvLsd*^*GFP*^ sample over wildtype. * We did not calculate a *p*-value for NvPHF21a-like due to a missing value in one of the control samples. **E** Co-IPs from *NvLsd1*^*GFP*^ planula with either anti-GFP Trap beads or control agarose beads followed by western blot analysis with indicated antibodies. This was repeated 3 times independently with the same result

Using a literature and homology-based approach we searched for core components of the CoREST complex in the genomes of a representative group of eukaryotes (see materials and methods and Additional file 1: Table S1). Lsd1 and HDAC1/2 are found across eukaryotes and are thought to have been present in the last common eukaryotic ancestor [10, 52–55]. CoREST orthologs, on the other hand, have a more complex evolutionary history. We find that definitive CoREST orthologs containing all three diagnostic domains, i.e., proteins with two SANT domains and an ELM2 domain, exist only in animals (54/54 species searched) and in choanoflagellates (5/22 species searched) (Additional file 1: Table S1). We do, however, find related sequences in other eukaryotic groups which, in many instances contain only one SANT domain connected to an ELM2 domain. Using domain-specific phylogenies, we show that the choanoflagellate CoREST SANT and ELM2 domains cluster with the respective domains from animal CoREST proteins (Additional files 2 and 3: Fig. S1 and 2) making it unlikely that these evolved from independent domain duplications. Instead, we conclude that bona fide CoREST proteins evolved before the last common ancestor of animals and choanoflagellates from a more ancestral CoREST-like protein possessing only a single SANT domain (Fig. 1A). In addition, similar to a recent preprint [56], we find CoREST sequences in plants by Blast search, but our analysis shows these to be contaminations from insects/mites. Firstly, in the cases where a genome for the plants is available, we do not find the sequences in those genomes, and secondly, phylogenetic analysis of the remaining sequences shows that they most likely represent arthropod sequences (Additional file 4: Fig. S3).

Based on the presence of all three core components in non-bilaterian animals we hypothesized that the CoREST complex predates the cnidarian-bilaterian split. To test this, we used an unbiased approach to identify interactors of NvLsd1. We took advantage of our previously characterized line in which we have endogenously tagged NvLsd1 with eGFP using CRISPR-Cas9 [51]. We used co-immunoprecipitation (Co-IP) with the GFP-Trap system coupled to liquid chromatography-mass spectrometry (LC–MS) to identify proteins that interact with NvLsd1 at the planula stage. We found that both NvCoREST and NvHDAC1/2 were highly enriched in the NvLsd1-GFP sample (Fig. 1C, D and Additional file 1: Table S2) as was GFP (~ ninefold upregulation). In addition, we found two other proteins, NvHMG20-like and NvPHF21a-like, putative *Nematostella* orthologs of HMG20A/B (iBRAF/BRAF35) and PHF21A (BHC80), which have also been identified as components of the vertebrate CoREST complex [15, 57–59] (Fig. 1B–D). In the case of NvPHF21a-like, we were unable to use a Student’s *t*-test as there was a missing value in one of the samples (i.e., making *n* = 2 and therefore not suitable to perform a Student’s *t*-test with all valid values). This could indicate that there was too little NVPHF21a-like in the sample to be detected. We are, however, confident that this is a bona fide interactor because it has a very high and consistent fold change in the two replicates for which we have values for both *NvLsd1*^*GFP*^ and control Co-IPs (Additional file 1: Table S2) (10.29 and 10.66 in the two replicates) and as it was among the most highly enriched proteins in a pilot experiment (along with the other four proteins shown here) (Additional file 5). As CoREST is an essential, core component of the complex we decided to investigate NvCoREST further. We generated an antibody against amino acids 1–199 of NvCoREST (Additional file 6: Fig. S4A). This antibody recognizes two bands by western blot (Additional file 6: Fig. S4B) which correspond approximately in size to two splice isoforms of *NvCoREST* which we can detect by PCR and which we have cloned and sequenced (Additional file 6: Fig. S4C). IP and western blot showed that NvLsd1 interacts with both isoforms of NvCoREST (Fig. 1E). Together this data shows that the CoREST complex is indeed present in *Nematostella* and contains the same subunits as present in vertebrates.

### NvCoREST expression is high in differentiated neural cells

**Fig. 2:**
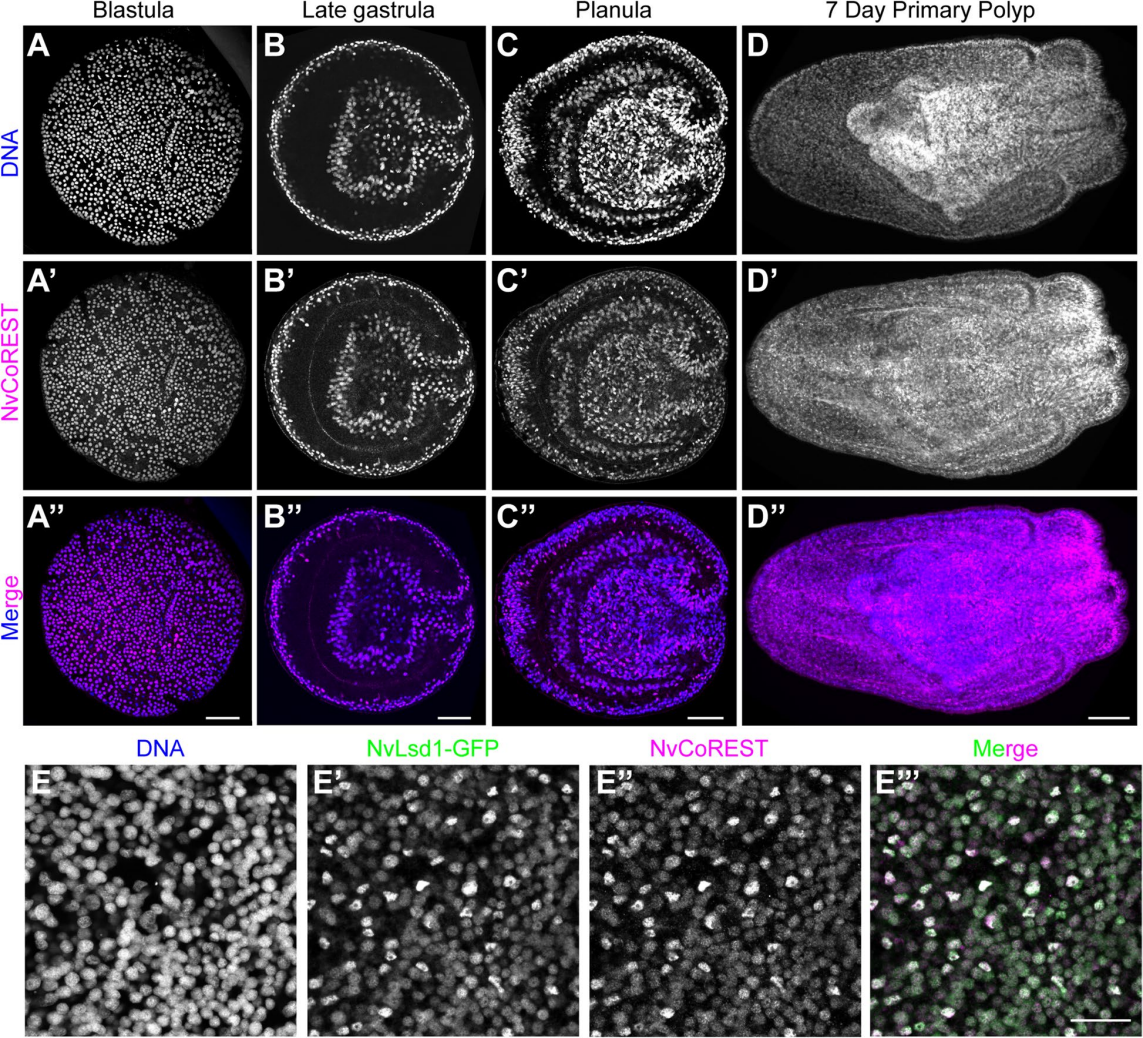
NvCoREST is ubiquitously expressed throughout development. **A**–**D** Confocal images of immunofluorescence staining showing NvCoREST localization throughout development. Stages are shown on top. **E** Close up of the ectoderm at planula stage showing colocalization of NvLsd1-GFP and NvCoREST. NvCoREST is shown in magenta, DNA in blue, and NvLsd1-GFP in green. All stainings were performed at least two times independently with a minimum of 10 embryos imaged per stage, per replicate with the same results. Scale bars: 50 µm (**A**–**D**) and 20 µm (**E**)

**Fig. 3:**
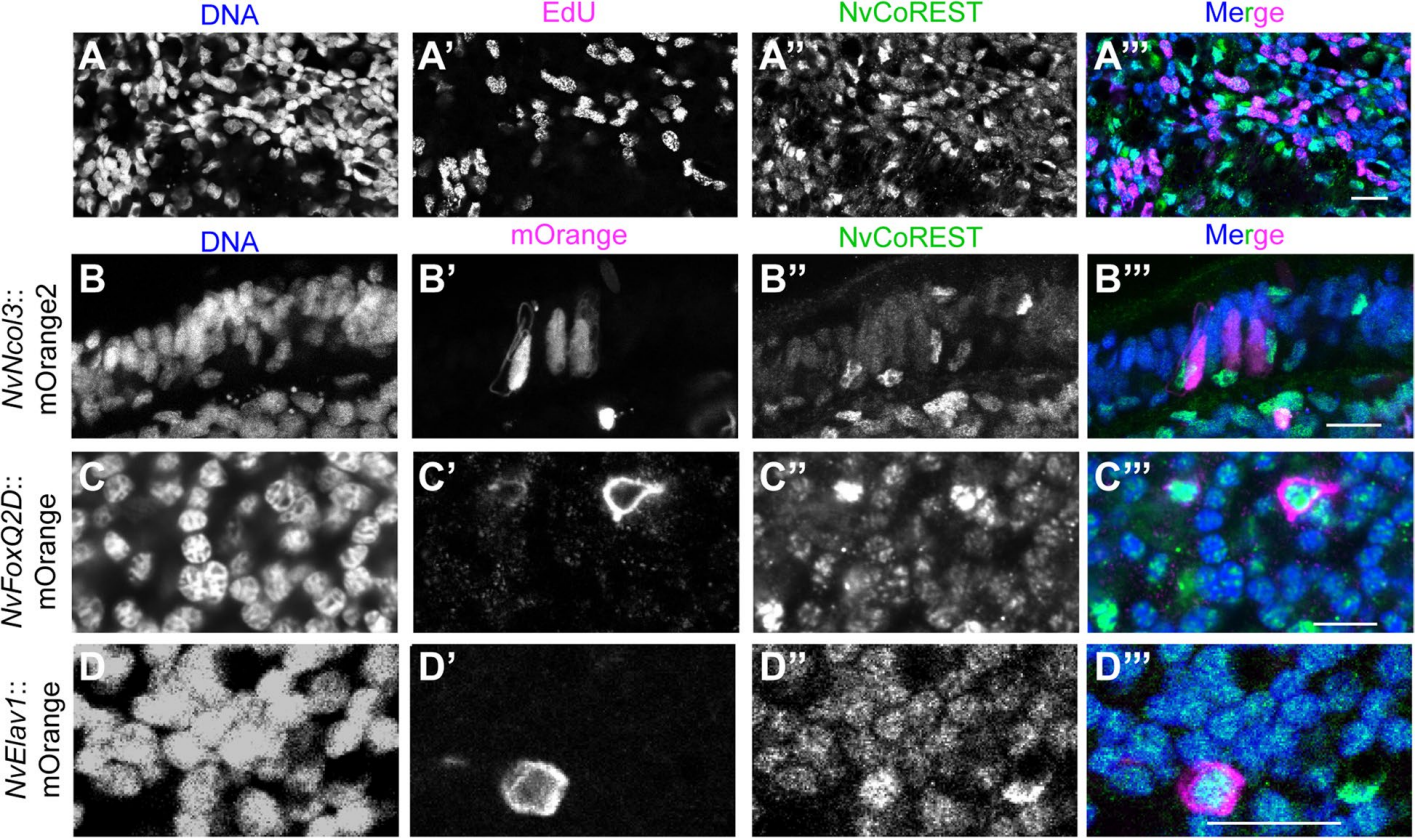
NvCoREST is low in proliferating cells and high in differentiated neural cells. **A** Confocal images of planula incubated with EdU for 30 min followed by immunofluorescence staining and Click-iT EdU detection. NvCoREST is shown in green, Click-iT EdU in magenta, and DNA in blue. **B**–**D** Confocal images of planula-stage transgenics stained for mOrange (magenta), NvCoREST (green), and DNA (blue) Transgenic lines used are indicated on the left. Stainings were performed two times independently with a minimum of 10 embryos imaged per genotype, per replicate with the same results. Scale bars: 10 µm

Having established that the CoREST complex is present in *Nematostella* we next wanted to understand how NvCoREST is expressed. Using immunofluorescence staining, we see that NvCoREST is ubiquitous and present in every nucleus at every stage studied except for mitotic nuclei from which it is excluded (Fig. 2A–D and Additional file 7: Fig. S5). The levels of NvCoREST are, however, heterogeneous and this heterogeneity appears gradually during development (Additional file 7: Fig. S5). Immunofluorescence staining in the NvLsd1-GFP line revealed that cells with higher levels of NvCoREST also have higher levels of NvLsd1-GFP (Fig. 2E). We have previously shown that NvLsd1 levels are high in differentiated neural cells but not their progenitors [51]. We also show this here for NvCoREST using immunofluorescence staining in parallel with EdU labeling for proliferating cells/progenitors and by immunofluorescence staining in three different neural reporter lines: *NvNcol3*::mOrange2 which labels cnidocytes, a cnidarian-specific neural cell type [60], *NvFoxQ2d*::mOrange which labels sensory cells [61] and *NvElav1*::mOrange which labels a large proportion of sensory cells and ganglion neurons [62]. We find that NvCoREST is relatively low in proliferating cells and high in differentiated neural cells (Fig. 3). Together this shows that NvCoREST expression is fully overlapping with that of NvLsd1 and is high in differentiated cells of the nervous system relative to their progenitors.

### *NvCoREST* is essential for *Nematostella* development

**Fig. 4:**
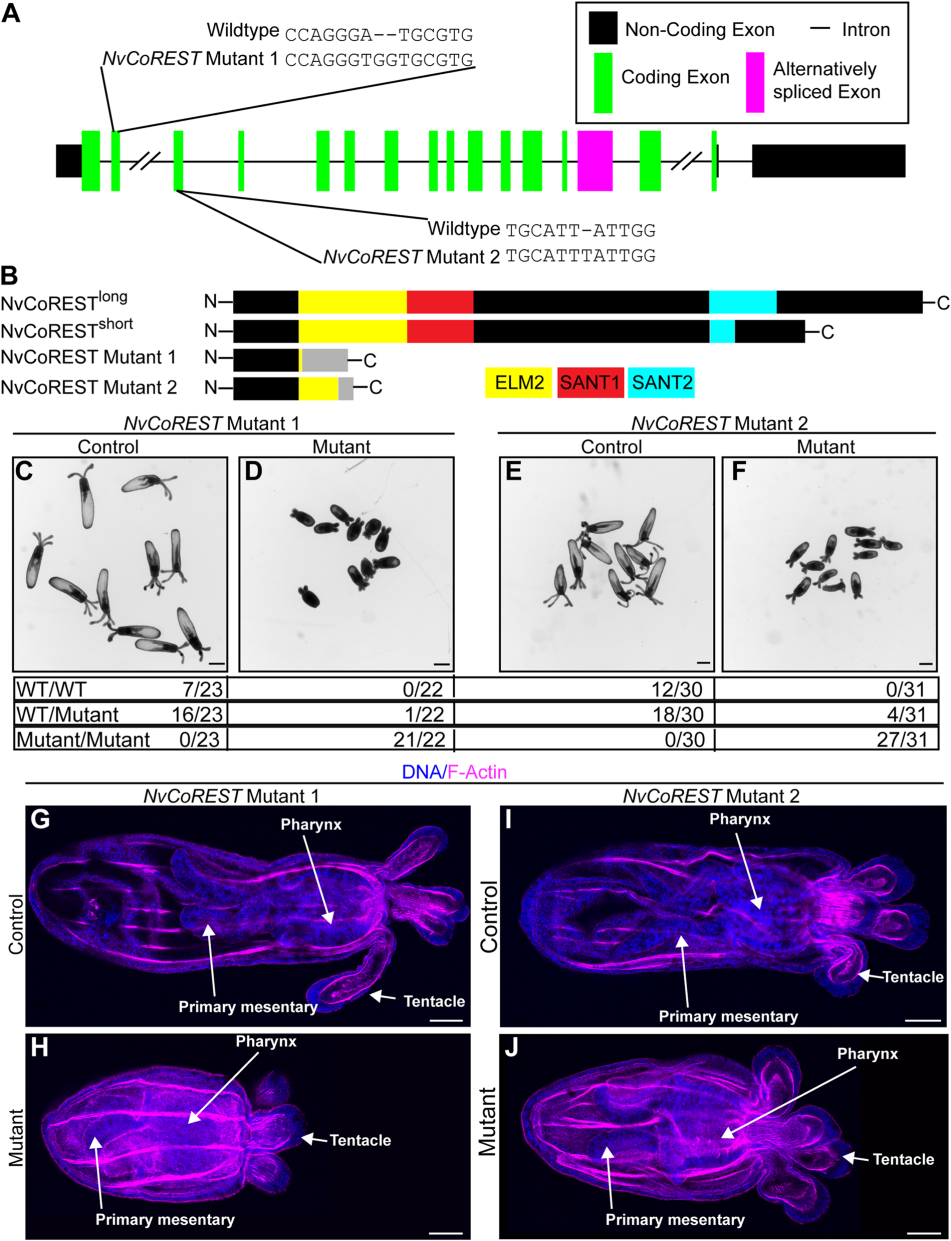
*NvCoREST* is required for normal development in *Nematostella*. **A** Schematic showing the intron–exon structure of *NvCoREST*, the location of the alternatively spliced exon, and indicating both the position and sequence of mutations in *NvCoREST* Mutant 1 and Mutant 2 lines. **B** Diagram representing the domain architecture of the NvCoREST long and short isoforms and the truncated products predicted based on the mutations. **C**–**F** Live images of primary polyp stage animals generated from in-crosses of heterozygous *NvCoREST* Mutant 1 or Mutant 2 animals, separated based on size phenotype (indicated on top). Below the images is sequencing data showing the number of animals with the indicated genotype found via sequencing within these pools of animals. Numbers are combined data from 4 independent replicates. **G**–**J** Confocal images of representative animals from each phenotypic group stained with phalloidin for F-actin (magenta) and DNA (blue). Stainings were performed three times, independently with a minimum of 10 embryos imaged per condition, per replicate with the same results. Scale bars: 200 µm (**C**–**F**) and 50 µm (**G**–**J**)

Next, to understand the function of *NvCoREST*, we generated two independent mutant lines using CRISPR-Cas9 targeting two different exons. Mutant 1 is an A to TGG substitution in exon 2 and mutant 2 harbors an insertion of a T in exon 3 (Fig. 4A). Both changes in the reading frame lead to premature stop codons and are predicted to generate early truncations of the NvCoREST protein leading to loss of both the long and short isoforms of NvCoREST (Fig. 4B). To assess the effects of loss of NvCoREST we in-crossed heterozygous animals from both lines, separately (Additional file 8: Fig. S6A). We did not see any difference in the overall survival of CoREST^−/−^ embryos (representing ~ 23% of planula larva) (Additional file 8: Fig. S6B). We then grew these animals to the primary polyp stage. At this stage we noted that the animals displayed either normal morphology or showed a severe size defect (Fig. 4C–F and Additional file 8: Fig. S6C). We can separate the animals based on this phenotype and using sequencing we see that most animals sorted as having this phenotype are homozygous mutant (hereafter referred to as mutant) and, importantly, animals exhibiting a wild-type phenotype are never homozygous mutants (hereafter referred to as control) (Fig. 4C–F). The mutant phenotype was seen in approximately 29% of animals, representing close to mendelian ratios (Additional file 8: Fig. S6D). We also performed immunofluorescence staining for NvCoREST and we do not see any nuclear staining in mutant animals from either line (Additional file 9: Fig. S7). In neither case were we able to find homozygous animals at the juvenile or adult stage showing that they do not survive past this stage. A more detailed morphological analysis shows that despite the overall growth defect, mutant animals have metamorphosed and generated the normal structures expected to be present at this stage, i.e., four tentacles, pharynx, and two primary mesenteries (Fig. 4G–J). Overall, this data shows that *NvCoREST* is required for normal development in *Nematostella*.

### Cnidocyte differentiation requires *NvCoREST*

**Fig. 5:**
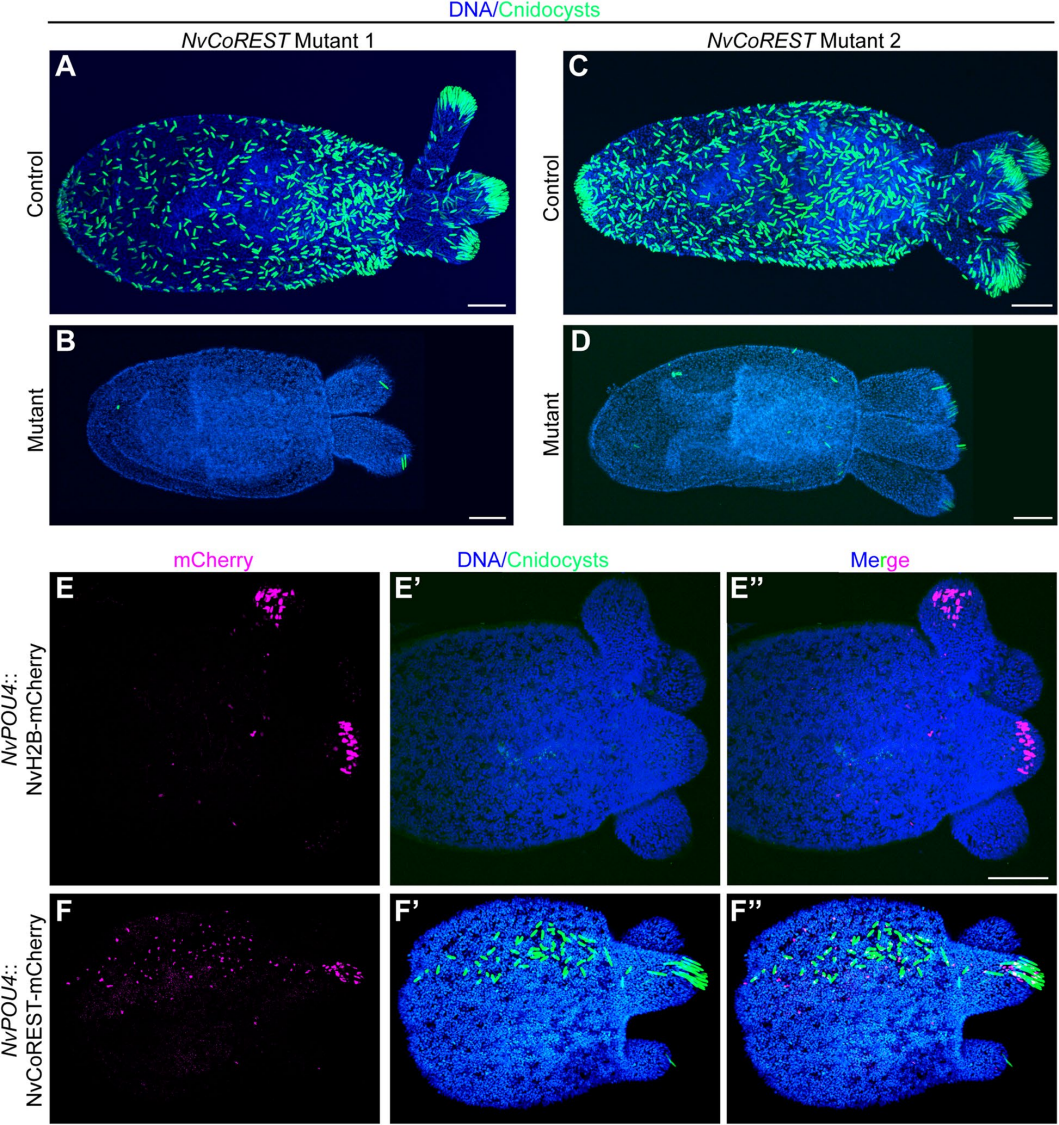
Loss of *NvCoREST* leads to a defect in cnidocyte differentiation. **A**–**D** Confocal images of control or mutant primary polyps from *NvCoREST* Mutant 1 or 2 showing cnidocysts (green) and DNA (blue). The mutant line is shown on top and the genotype to the left. Stainings were performed three times independently with the same results with a minimum of 10 embryos imaged per condition, per replicate. **E**,** F** Confocal images of immunofluorescence staining on mutant primary polyps from *NvCoREST* Mutant 1 line showing mosaic patches of NvPOU*4*::H2B-mCherry (**E**) or *NvPOU4*::NvCoREST-mCherry (**F**) expression stained with anti-DsRed antibody for mCherry (magenta), DAPI for cnidocysts (green), and DNA (blue). Data was collected across two independent biological replicates and *n* = 10 for *NvPOU4*::H2B-mCherry and *n* = 19 for *NvPOU4*::NvCoREST-mCherry of which 10/10 and 18/19, respectively, had the observed phenotype. Scale bars: 50 µm

We next looked at the effect of loss of *NvCoREST* on cnidocytes given that we have previously shown that *NvLsd1* is essential during cnidocyte differentiation. Cnidocytes are highly specialized, cnidarian-specific neural cells used for prey capture and defense and contain a specialized organelle, the cnidocyst, which contains a coiled thread that can be explosively discharged and acts like a harpoon [63]. In both mutant lines, we see that loss of *NvCoREST* leads to an almost complete loss of differentiated cnidocytes as determined using a protocol that utilizes DAPI to label the mature cnidocyst (Fig. 5A–D) [64, 65]. We also wanted to assess whether the reduction in cnidocyte number was due to a role for *NvCoREST* in the specification or later differentiation of cnidocytes. To do so we performed immunofluorescence staining for NvNcol3, a protein found in the cnidocyst. The epitope recognized by this antibody, however, is only available prior to the maturation of the cnidocyst and thereby acts as a marker for earlier stages of cnidocyte differentiation [66]. We found that there was abundant NvNcol3 staining in the *NvCoREST* mutants (Additional file 10: Fig. S8). The NvNcol3 staining, however, did not show the regular elongated capsules that are visible in the controls (arrows in Additional file 10: Fig. S8A’-A’’). This indicates that cnidocytes were still specified in the absence of *NvCoREST* but could not complete the differentiation process. In the case of *NvLsd1*, we have previously shown that the requirement during cnidocyte differentiation is cell autonomous as the phenotype can be rescued by re-expression of *NvLsd1* using *NvPOU4* regulatory elements that drive expression predominantly in cnidocytes in the ectoderm [51, 67]. We performed the same analysis here using a *NvPOU4::NvCoREST-mCherry* plasmid. We find that in almost all cases (18/19) where we saw mosaic patches expressing NvCoREST-mCherry, we also see a rescue of the cnidocyte phenotype, something we do not see when we express NvHistone2B-mCherry as control (Fig. 5E, F). Unfortunately, we were unable to achieve expression of a rescue construct encoding the shorter splice variant of *NvCoREST*. Finally, we have also shown that loss of *NvLsd1* results in a phenotype in the *NvElav1*::mOrange^+^ nervous system characterized by a modest disorganization of the nerve net, the appearance of numerous mOrange^+^ puncta, and expansion of the mOrange signal into surrounding epithelial cells. We have also looked here at the *NvElav1*::mOrange^+^ nervous system by crossing the *NvElav1::mOrange* transgene into the background of *NvCoREST* mutant 1. When we compare control and mutant animals, we see a similar, but less severe effect as in *NvLsd1* mutants (Additional file 11: Fig. S9). Together this shows that NvCoREST is essential for the post-mitotic differentiation of cnidocytes while being largely dispensable for the formation of the *NvElav1*::mOrange^+^ nervous system.

## Discussion

We show here that CoREST is specific to animals and choanoflagellates, that the CoREST complex is present in *Nematostella* and that it is required during development and for the differentiation of cnidocytes. These observations provide a plausible explanation as to how the more ancient complex members, Lsd1 and HDAC1/2, may have been co-opted during evolution to play roles in development. The integration of these two chromatin modifiers into the CoREST complex thus may have facilitated the coordinated regulation of chromatin at specific genomic loci.

We show that although bone fide CoREST proteins exist only in animals and choanoflagellates, related sequences are found in other clades. A recent report described CoREST sequences in plants but we show here that these sequences represent contaminations, most likely from arthropods. From our data, we hypothesize that CoREST evolved from an ancestral protein that contains only one SANT domain. Whether these CoREST-like proteins function in a similar manner to animal CoREST proteins is not clear. In animals, the SANT2 domain is required to stimulate the activity of Lsd1, while the interaction with Lsd1 is mediated via the linker region between the SANT domains [23, 24]. The other SANT domain, along with the ELM2 domain, is important for the interaction between CoREST and HDAC1/2 [16, 23] and possibly for nucleosome binding [22]. It is therefore possible that the ancestral CoREST-like sequence was capable only of interaction with HDAC1/2 or Lsd1 and not with both and that the ability to concomitantly bind both, and therefore form a CoREST/Lsd1/HDAC1/2 complex, evolved only in choanoflagellates and animals. Further investigation would be required to test the binding specificity of the non-animal CoREST-like proteins and determine if they interact with Lsd1, HDAC1/2, or both. In *Nematostella*, we have shown that there are two splice isoforms of NvCoREST, one of which has a partial deletion of the SANT2 domain. This protein is, however, able to bind NvLsd1, likely due to the intact linker region, and therefore, the function and role of this variant are hitherto unknown. Given that this splice variant lacks most of the SANT2 domain it is possible it may act as a dominant negative which can bind Lsd1 but not stimulate its activity, but this would need to be tested experimentally. Similarly, a splice variant of human RCOR3 (CoREST3) lacks the SANT2 domain and cannot stimulate LSD1 activity [35]. It has also been shown that *Drosophila* CoREST has several splice isoforms including one which lacks the SANT2 domain entirely, although again the functional consequences of this are unknown [68]. Another *Drosophila* spice variant contains a large insertion in the linker domain which abolishes binding to Lsd1 [29].

Our data point to a strong functional connection between NvLsd1 and NvCoREST. Not only do they interact but both proteins are also expressed in precisely the same manner and the phenotypes of loss of *NvCoREST* and *NvLsd1* are highly similar regarding the size of the animals as well as the loss of cnidocytes [51]. We consider this as strong support for the hypothesis that most of the roles of NvLsd1 in *Nematostella* are mediated through the NvCoREST complex. A similar picture has also emerged in bilaterians where Lsd1 and CoREST interact, co-localize, and indeed phenocopy each other in multiple systems [15, 29–31, 41, 43]. In the future, it will be useful to interrogate H3K4 methylation in different cell types in *Nematostella* in the presence or absence of NvLsd1 and NvCoREST in order to refine the precise molecular basis of these phenotypes and directly compare the phenotypes by, for example, comparing all differentially expressed genes.

There are, however, differences between the two loss-of-function phenotypes. The size defect observed in *NvCoREST* mutants appears to be more severe than that seen in *NvLsd1* mutants, though it is possible that these differences result from the use of non-isogenic animals for generating the mutants. Secondly, we do not see the same strong effects in *NvCoREST* mutants on the *NvElav1*::mOrange^+^ nervous system as in *NvLsd1* mutants. Attempts to quantify this defect (in both NvLsd1 and NvCoREST mutants) have thus far proved difficult and therefore this effect remains purely qualitative. For this reason, we cannot say with certainty what effect, if any, loss of *NvCoREST* has on the *NvElav1*::mOrange^+^ nervous system. Despite this uncertainty, this may indicate that some functions of *NvLsd1* are not mediated through the NvCoREST complex. This is interesting as roles outside of the CoREST complex have been assigned for NvLsd1 in mammals, particularly as a transcriptional activator e.g., in the mammalian nervous system [69–72]. It will therefore be interesting in the future to more deeply analyze the function of *NvLsd1* and *NvCoREST* in the *NvElav1*::mOrange^+^ nervous system and the development of tools for cell type-specific loss of function will greatly help in this regard.

Though the CoREST proteins of choanoflagellates and animals have a common evolutionary origin, they have been lost repeatedly from choanoflagellates, but in none of the animal genomes that we analyzed (Additional file 1: Table S1). Despite potential bias introduced by the search strategy and the possibility for sequence divergence to mask orthology, this indicates that an essential function of CoREST evolved only in animals. Even within animals, it is possible that CoREST first had a different function and only later became the scaffold of the CoREST complex as it exists in bilaterians. We have shown here that at least in the cnidarian-bilaterian ancestor this function was already present. Using our unbiased approach, we have shown that not only the three core components CoREST, Lsd1, and HDAC1/2 are present in the *Nematostella* CoREST complex, but homologs of the two additional components of the vertebrate CoREST complex, PHF21A, and HMG20A/B, are also present. A recent report on CoREST-containing complexes in *Drosophila* did not uncover any related proteins as major complex constituents [29]. This suggests that the complex present in *Nematostella* is highly similar to that found in vertebrates and may have maintained some ancestral features that have been lost in other lineages. We have similarly recently shown that *Nematostella* contains a more vertebrate-like Polycomb Repressive Complex 1 (PRC1) repertoire at the level of presence/absence of complex specific components [73]. Together this suggests that anthozoan cnidarians and vertebrates may have retained more components of an ancestral machinery for chromatin regulation than other lineages and that *Nematostella* therefore is a useful model system in which to dissect fundamental aspects of the biology of such complexes.

## Conclusions

We have shown here that a vertebrate-like CoREST-Lsd1-HDAC1/2 complex evolved early in animal evolution and is required for differentiation of a neural cell type in *Nematostella*. Our observations suggest that the evolution of multiprotein chromatin modification complexes is one mechanism that contributed to the elaboration of gene regulatory programs involved in cell type differentiation in early animals.

## Methods

### Animal care and maintenance

*Nematostella* were maintained at 18–19 °C in 1/3 filtered seawater [*Nematostella* medium (NM)] and spawned as described previously [74]. Fertilized eggs were removed from their jelly packages by incubating in 3% cysteine in NM for 20 min followed by extensive washing in NM. Embryos were reared at 21 °C and were fixed at 12 h post fertilization (hpf) (blastula), 16 hpf (late blastula), 20 hpf (early gastrula), 30 pfh (late gastrula), 72 hpf (planula), or at 13 days (primary polyp).

### Orthology search

The presence/absence of orthologs of Lsd1 and HDAC1/2 in different groups was extracted from existing literature [10, 52–55]. The presence of choanoflagellate Lsd1 orthologs was assessed by reciprocal blast searches in the genomes of *Salpingoeca rosetta* and *Monosiga brevicollis.* Homologs of HMG20a/b and PHF21a were identified by reciprocal blast search in the *Nematostella* genome using human HMG20a (UniProt: Q9NP66) and PHF21a (UniProt: Q96BD5), respectively as a query. CoREST homologs were searched by BlastP and TBlastN through the NCBI BLAST interface on available proteins, genomes, and transcriptomes as well as on dedicated databases (see Additional file 1: Table S1) [75–77], using the human CoREST1, *Nematostella vectensis* CoREST, and *Chromosphaera perkinsii *CoREST-like sequences as queries. Proteins were considered as putative CoREST-like if they included successive ELM2 and SANT domains. The analyzed genomes are listed in Additional file 1: Table S1 and the accession numbers are given in Additional files 2, 3 and 4: Fig. S1-3. ELM2 and SANT domains were then extracted manually from the retrieved sequences and aligned with a selection of ELM2 and SANT domain sequences obtained from the PFAM database. ELM2, SANT and CoREST metazoan proteins were each aligned using the MAFFT v7.271 L-INS-I algorithm [78]. Neighbor-joining analyses including bootstrap analyses (500 replicates) were performed using MEGA v7 [79].

### Immunoprecipitation

Embryos from either wildtype or NvLsd1^GFP/GFP^ crosses were grown until the planula stage. Approximately 50 μl of planula (volume of tissue without media) were used per IP. They were placed in lysis buffer (10 mM TricHCl pH 8, 150 mM NaCl, 2 mM EDTA, 1% NP40, 10% glycerol) with cOmplete EDTA-free Protease Inhibitor Cocktail (Roche, 4,693,159,001) and homogenized by passing through a 27-G needle. Samples were then incubated on ice for 30 min and mixed approximately every 5 min by passing through the needle. Samples were then centrifuged at full speed for 15 min and 600 μl of supernatant was used for IP. For each sample, 30 μl GFP-Trap Agarose or Binding Control Agarose Beads (Chromotek, gta-10, and bab-20) were washed once in dilution buffer (10 mM TricHCl pH 7.5, 150 mM NaCl, 0.5 mM EDTA) and then spun at 2500 g for 2 min. The lysate was diluted with 900 μl dilution buffer and then added to the beads. This was incubated at 4 °C for 2 h rotating. Following this, the beads were washed at least 6 times in 1 ml wash buffer (Dilution buffer + 0.5% NP40) for > 10 min each at 4 °C. In the final wash, the beads were moved to a new tube. When protein was used for LC–MS analysis the wash buffer was removed and the beads were resuspended in 100 μl MilliQ H_2_O and frozen at − 80 °C until being processed further. In the case of western blotting the beads were incubated in 2X Laemmli sample buffer (0.1 M TrisHCl pH 6.8, 2% SDS, 20% glycerol, 4% β-mercaptoethanol, 0.02% bromophenol blue) at 95 °C, spun down and the supernatant was used for further analysis.

### Sample preparation for liquid chromatography-mass spectrometry (LC–MS)

Beads were thawed to room temperature (RT) and centrifuged at 2500 g for 2 min and the H_2_O was removed. The beads were then resuspended in 40 μl trypsin buffer (50 mM Tris, 1 mM CaCl_2_, pH8), 4 μl of 0.1 M DTT was added, and the samples were heated to 95 °C for 5 min. The samples were then cooled to RT and 5 μl of 200 mM iodoacetamide was added, and the samples were incubated, shaking at RT for one hour. 0.8 μl of 0.1 M DTT was added to quench the remaining iodoacetamide, and samples were incubated shaking for 10 min. The pH was adjusted to approximately pH8 with 0.5 M Tris, 2 μg of Trypsin (Promega, V5111) was added to each sample and they were incubated shaking at 37 °C overnight (o/n). Following this, 5 μl of 10% trifluoroacetic acid was added to each sample and the peptide solutions were cleaned up with an Oasis HLB µElution plate (2 mg sorbent; Waters). Following elution, samples were frozen at − 80 and freeze-dried.

### LC–MS analysis

Preliminary studies with samples containing 0.8 µg tryptic peptides dissolved in 2% acetonitrile (ACN) and 0.5% formic acid (FA) were injected into an Ultimate 3000 RSLC system coupled to a Q Exactive HF mass spectrometer (Thermo Scientific, Waltham, MA, USA). The MS1 resolution was 120,000 and the scan range 375–1500 m/z, the AGC target was set to 3e^6^ and the maximum injection time was 100 ms The intensity threshold was set at 5.0e^4^ and dynamic exclusion lasted for 20 s. The MS/MS scans consisted of HCD with normalized collision energy at 28, quadrupole isolation window at 1.6 m/z, and Orbitrap resolution at 15,000.

For the final experiments, control and *NvLsd1*^*GFP*^ samples containing the same amount of peptide were analyzed in an Orbitrap Eclipse Tribrid mass spectrometer equipped with an EASY-IC/ETD/PTCR ion source and FAIMS Pro interface (Thermo Scientific, San Jose, CA, USA). The MS1 resolution and the scan range were set as above, AGC target was set to standard, maximum injection time was automatic and RF lens at 30%. The intensity threshold was also at 5.0e^4^ and dynamic exclusion lasted for 30 s. The MS/MS scans consisted of HCD with collision energy at 30%, quadrupole isolation window at 4 m/z, and Orbitrap resolution at 30,000. FAIMS was set up with the standard resolution mode and a total gas flow of 4.6 L/min. The CVs were set to − 45 and − 65 V. The mass spectrometry proteomics data have been deposited to the ProteomeXchange Consortium via the PRIDE [80] partner repository with the dataset identifier PXD033068.

### Statistical and bioinformatic analyses

The LC-Q Exactive raw files were searched in MaxQuant (version 1.6.14.0, Max Planck Institute for Biochemistry, Martinsread, Germany) [81] and the spectra were searched against the nveGenes.vienna database version 2008_02 (https://figshare.com/articles/dataset/Nematostella_vectensis_transcriptome_and_gene_models_v2_0/807696). The LC-Eclipse raw files were searched in Proteome Discoverer Software (version 2.5, Thermo Fisher Scientific, Bremen, Germany) using the SEQUEST HT database search engine with Percolator validation (FDR < 0.01), and against the uniprot-proteome UP000001593 database version 2021_02. In order to test for GFP within the samples, we added GFP to this database (UniProt ID: P42212). Perseus (version 1.6.15.0, Max Planck Institute for Biochemistry) [82] was used to process and normalize the data. Proteins with three valid values in each group were selected for statistical comparisons using *t*-test. Proteins with *p*-values < 0.05 were considered to have significantly different abundance. Analyzed data is provided in Additional file 5.

### Western blotting

Protein extraction was performed on Lsd^GFP^ or wild-type planula. Animals were placed in RIPA buffer (150 mM NaCl, 50 mM Tris pH8, 1% NP40, 0.5% DOC, 0.1% SDS) supplemented with cOmplete EDTA-free Protease Inhibitor Cocktail (Roche, 4,693,159,001) and homogenized by passing through a 27G needle. Samples were incubated on ice for 30 min and mixed by passing through the needle every 5 min and centrifuged at full speed for 15 min at 4 °C. The supernatant was kept and the protein concentration was quantified using the Qubit™ Protein Assay (Invitrogen, Q33212). Thirty micrograms of protein was used per lane, mixed 1:1 with 2X Laemmli sample buffer, and boiled for 5 min before loading. For IP samples, beads were boiled in 2 volumes 2 × Laemmli sample buffer for 5 min, spun down and the supernatant was loaded directly onto the gel. PageRuler™ Plus prestained protein ladder, 10 to 250 kDa (Thermo Scientific, 26,619) was used. SDS PAGE was performed using 7.5% or 4–20% Mini-PROTEAN® TGX™ precast protein gels (BIO-RAD, 4,561,023/4561094) run in running buffer (25 mM Tris, 192 mM Glycine, 0.1% SDS) at 100 V for ~ 120 min. Transfer was performed using Trans-Blot Turbo Mini 0.2 µm PVDF Transfer Pack (BIO-RAD, 1,704,156) on a Trans-Blot Turbo transfer system (BIO-RAD) using the high molecular weight program. After transfer, the membrane was washed in PBT (PBS + 0.1% Tween) several times and blocked with 5% milk powder in PBT (MPBT) at RT for 1 h. The blots were incubated o/n at 4 °C in 1° antibody in MPBT. The membranes were then washed several times in PBT and incubated in 2° antibody in MPBT at RT for 1 h. Membranes were then washed several times in TBT and the signal was revealed using Clarify ECL substrate (BIO-RAD, 1,705,060) and imaged on a ChemiDoc XRS + (BIO-RAD). The blots were then washed in PBT and blocked again in 5% MPBT for 1 h at RT. They were then incubated o/n at 4 °C with 1° antibody and processed as for the first antibody. Antibodies and dilutions are listed in Additional file 1: Table S4.

### Immunofluorescence

Animals at the planula stage and older were anesthetized with MgCl_2_ and then killed quickly by adding a small volume (20–30 μl/ml) of 37% formaldehyde directly into the media. They were then fixed in ice-cold 3.7% formaldehyde in PBTx [PBS(Phosphate Buffered Saline) + 0.2% Triton X-100] for 30–60 min (when staining for NvCoREST short fixations yield better staining) or for > 60 min or o/n (for all other antibodies) at 4 °C. Samples were washed > 4 times in PBTx at RT, blocked in Block (3% BSA/5% Goat serum in PBTx) for > 1 h at RT, and incubated in primary antibody diluted in Block o/n at 4 °C. Samples were then washed extensively in PBTx (> 5 washes for 2 h or more) at RT, blocked for 1 h at RT in Block, and incubated o/n or over the weekend in secondary antibody diluted in Block at 4 °C. If Phalloidin staining was performed, Alexa Fluor™ 488 or 633 Phalloidin (Thermo Fisher Scientific, A12379/A22284) was added here at 1:50–1:100. Samples were then incubated in Hoechst 33,342 (Thermo Fisher Scientific, 62,249) at 1:100 in PBTx for 1 h at RT followed by extensive washing in PBTx (> 5 washes for 2 h or more). Animals were mounted in ProLong™ Gold Antifade Mountant with DAPI (Thermo Fisher Scientific, P36935) and imaged on a Leica SP5 confocal microscope. Antibodies and dilutions are listed in Additional file 1: Table S4.

### EdU labeling

Animals used in Edu labeling experiments were incubated in 10 mM EdU in NM for the desired time and then treated for IF as described. After the final set of PBTx washes, EdU incorporation was visualized using the Click-iT™ EdU Imaging Kit with Alexa Fluor™ 488 or 647 (Thermo Fisher Scientific, C10337/C10337) following the manufacturer’s protocol. Samples were mounted and imaged as for IF.

### DAPI staining for cnidocysts and counting

DAPI staining for cnidocysts was performed as previously published [64, 65] with slight modifications. Animals were processed as for IF with the addition of 10 mM EDTA to all solutions. Following the final PBTx wash, the samples were washed twice with MilliQ H_2_O and then incubated in 200 μg/ml DAPI in milliQ H_2_O o/n at RT. The samples were then washed once with MilliQ H_2_O, twice with PBTx with 10 mM EDTA, and mounted and imaged as for IF.

### CRISPR-Cas9 injections and genotyping

sgRNA was produced using a template generated by primer annealing. A PCR was set up containing 5 μl of each primer (100 mM) (one sgRNA specific and one generic), 2 μl dNTPs (10 mM each), 2 μl Q5 polymerase (NEB, M0491), 10 μl Q5 reaction buffer and 31 μl H_2_O with the following protocol: 98 °C, 90 s; 55 °C, 30 s; and 72 °C, 60 s. This was purified using a PCR clean-up kit (Promega, A9281). The sgRNAs were synthesized using the MEGAscript™ T7 Transcription Kit (Invitrogen, AMB13345) including the DNase treatment, and were precipitated by adding 1:1 LiCl (7.5 M) (Invitrogen AM9480) and incubating at − 20 °C for 30 min followed by centrifugation at full speed at 4 °C for 15 min and extensive EtOH washes. The concentration was calculated using a Nanodrop. Primers are given in Additional file 1: Table S3. *NvCoREST* mutants were produced similarly to previously published [51, 83, 84]. Eggs were injected with a mix containing sgRNA (130 ng/μl), Cas9 (PNA Bio, CP01) (500 ng/μl), and 1:4 Dextran, Alexa Fluor™ 568 (Invitrogen, D22912) (200 ng/μl in 1.1 M KCl) that was incubated at 37 °C for 5–10 min prior to injection. Injected animals were raised to sexual maturity and crossed to wildtypes. Individual F1 offspring were analyzed by PCR and sequencing to identify F0’s carrying the desired mutations. To extract genomic DNA, individual primary polyps were placed in tubes, the NM removed and 100% EtOH added. After 5 min, this was removed, and the tubes were placed at 50 °C for 45 min to allow the remaining EtOH to evaporate. 50 μl genomic extraction buffer (10 mM Tris pH8, 1 mM EDTA, 25 mM NaCl, 200 μg/μl ProteinaseK) was added to each and incubated at 50 °C for 2 h and 98 °C for 15 min. 2 μl of this was used for PCR and sequencing. Primers are given in Additional file 1: Table S3. Once an F0 carrier was identified, the remaining F1 offspring from that carrier were genotyped using a piece of tissue to generate a pool of F1 heterozygous animals which were then crossed and the offspring of these crosses were used in experiments.

### Selection and analysis of homozygous mutants

To evaluate the survival of *NvCoREST* mutants, 20 embryos from Mutant 1 heterozygous in-crosses were separated after fertilization and grown until planula larva (approximately 72 h). This was repeated twice with 17 and 18 embryos surviving in each experiment. These embryos were then genotyped as described above. For analysis of the numbers of animals at the primary polyp stage, between 30 and 40 embryos were grown until 13 days and then separated based on phenotype, imaged, and genotyped individually. The numbers counted are lower as some animals die prior to this stage, both in mutants and in wild-type animals.

### Cloning

For generating cDNA for PCR, RNA was extracted as previously published [51]. The SuperScript™ III first-strand synthesis system (Invitrogen, 18,080,051) was used to generate cDNA. All PCRs were performed with Q5 polymerase and primers are listed Additional file 1: Table S3. For cloning of the *NvCoREST* cDNA (Figure S2) the fragments were cloned using the CloneJET PCR Cloning Kit (Thermo Fisher Scientific, K1231). To generate the NvPOU4::NvCoREST-mCherry construct the backbone was amplified from the NvPOU4:: mCherry construct [67] and the CoREST open reading frame from cDNA, respectively. Assembly of the construct was done using the NEBuilder® HiFi DNA Assembly master mix (NEB, E2621). 

### Transgenesis

In order to generate F0 mosaic transgenics, we used I-SceI mediated transgenesis as previously described [85] with minor modifications. Eggs were injected with a mix containing: plasmid DNA (10 ng/μl), ISceI (1U/μl) (NEB, R0694), Dextran Alexa Fluor™ 568 (100 ng/μl), and CutSmart buffer (1 ×). The mix was incubated for 30 min at 37 °C before injection.

## Supplementary Information

**Additional file 1:** **Table S1-4.** **Table S1.** Search for COREST-like in non-metazoan and metazoan genomes and transcriptomes. **Table S2.** Data from individual replicates of LC-MS experiments. **Table S3.** List of primers used in this study. **Table S4.** List of antibodies and dilutions.

**Additional file 2:** **Fig S1.** Neighbor-joining phylogeny of the SANT domain, including a selection of SANT domain sequences from various protein families, as well as metazoan and non-metazoan CoREST-like sequences.

**Additional file 3: Fig S2.** Neighbor-joining phylogeny of the ELM2 domain, including a selection of ELM2 domain sequences from various protein families, as well as metazoan and non-metazoan CoREST-like sequences.

**Additional file 4: Fig S3.** Neighbor-joining phylogeny of metazoan CoREST full protein. Bootstrap values are indicated next to the nodes. The tree is rooted with poriferan and ctenophore sequences. The distance scale represents the percentage of genetic or nucleotide variation between the sequences. The 4 “Plant CoREST-like” sequences retrieved from the NCBI TSA database group are likely arthropod contaminations as they cluster with insect and acharian CoREST sequences.

**Additional file 5.** Analyzed mass spec data.

**Additional file 6: Fig S4.** NvCoREST isoforms and antibody validation. (A) Alignment of full length Nematostella CoREST with human CoREST1 (UniProt: Q9UKL0). Alignment was performed using Clustal Omega [86]. Conserved domains are highlighted with coloured boxes; ELM2 in yellow, SANT1 in green and SANT2 in cyan. The portion of the protein used to generate the NvCoREST antibody is outlined with a red box. The alternatively spliced exon is outlined with a black box. (B) Western blot using anti-NvCoREST antibody showing two bands corresponding in size to the expected sizes of the full length and short isoform of NvCoREST, shown in the box at the bottom. Protein was extracted at planula stage. (C) PCR analysis using primers to amplify full length NvCoREST from cDNA from different developmental stages. The stage from which the cDNA was generated is shown on top measured in hours post fertilization (hpf). The expected sizes of full length and the short isoform of NvCoREST are shown in a box. Three bands are present; the highest and lowest correspond to the full length and short isoform of NvCoREST, respectively, and were successfully closed and sequenced. The middle band was never cloned and is presumably a PCR artifact, likely due to hybridization between the full length and short isoforms. Western blot and PCR analysis were carried out two times, independently with the same results.

**Additional file 7: Fig S5.** The heterogeneity in NvCoREST levels appears over developmental time. (A to C) Confocal images of immunofluorescence staining performed on early embryos. Stages used are indicated to the left of the images. (A’’’ to C’’’) show close ups. Staining’s were performed two times independently with a minimum of 10 embryos imaged per replicate with the same results. Scale bars: 20 µm.

**Additional file 8: Fig S6.** Additional data on NvCoREST mutants. (A) Schematic representation of the crosses used when analyzing NvCoREST mutants. (B) Genotyping results on larva derived from NvCoREST mutant 1 in-crosses at planula larva stage. The planulae showed no visible phenotype and were selected at random and genotyped by PCR and sequencing. (C) Brightfield image of live primary polyps derived from an in-cross of heterozygous NvCoREST mutant 1 animals. The animals displaying the mutant phenotype are highlighted with a white circle. (D) Data showing the number of animals with mutant or wildtype phenotype from 4 independent replicates. For each experiment 20-30 eggs were selected and grown to primary polyp stage and then those surviving were separated into either wildtype or mutant categories and counted.

**Additional file 9: Fig S7.** NvCoREST staining is absent in both NvCoREST mutant lines. (A to D) Confocal images of immunofluorescence staining on mutant and control primary polyps from NvCoREST Mutant 1 or 2 lines stained for NvCoREST (magenta) and DNA (blue). Mutant line and genotype are shown to the left. Ubiquitous nuclear NvCoREST staining can be seen in control but is absent in mutant animals while non-specific staining of actin filaments can be seen in both. Stainings were performed two times independently with a minimum of 10 embryos imaged per genotype, per replicate with the same results. Scale bars: 20 µm.

**Additional file 10: Fig S8.** NvCoREST mutants still express NvNcol3. (A and B) Confocal images of immunofluorescence staining on NvCoREST Mutant 1 primary polyps showing NvNcol3 in Magenta, DNA in blue and cnidocysts in Green. Arrows in A’ and A’’ indicate developing cnidocysts with normal morphology. The experiment was performed twice, independently and a minimum of 10 animals per genotype, per replicate were analyzed and showed the same result. Scale bars: 50 µm.

**Additional file 11: Fig S9.** Loss of NvCoREST does not affect the NvElav1::mOrange^+^ nervous system. (A and B) Confocal images of immunofluorescence staining on control and mutant primary polyps showing DNA in blue and *NvElav1*::mOrange in magenta. Heterozygous NvCoREST mutant 1 animals were crossed to animals double heterozygous for NvCoREST mutant 1 and the *NvElav1*::mOrange transgene. The experiment was performed three times, independently and a minimum of 10 animals per genotype, per replicate were analyzed and showed the same result. Scale bars: 50 µm.

## Data Availability

All data necessary to reproduce the results are available in the main text or the supplementary materials. The mass spectrometry proteomics data have been deposited to the ProteomeXchange Consortium via the PRIDE [80] partner repository with the dataset identifier PXD033068. Novel reagents and animal lines are freely available upon request.

## References

[1] Putnam NH, Butts T, Ferrier DE, Furlong RF, Hellsten U, Kawashima T, Robinson-Rechavi M, Shoguchi E, Terry A, Yu JK (2008). The amphioxus genome and the evolution of the chordate karyotype. Nature.

[2] Putnam NH, Srivastava M, Hellsten U, Dirks B, Chapman J, Salamov A, Terry A, Shapiro H, Lindquist E, Kapitonov VV (2007). Sea anemone genome reveals ancestral eumetazoan gene repertoire and genomic organization. Science.

[3] Ryan JF, Pang K, Schnitzler CE, Nguyen AD, Moreland RT, Simmons DK, Koch BJ, Francis WR, Havlak P, Program NCS (2013). The genome of the ctenophore Mnemiopsis leidyi and its implications for cell type evolution. Science.

[4] Simakov O, Marletaz F, Cho SJ, Edsinger-Gonzales E, Havlak P, Hellsten U, Kuo DH, Larsson T, Lv J, Arendt D (2013). Insights into bilaterian evolution from three spiralian genomes. Nature.

[5] Srivastava M, Begovic E, Chapman J, Putnam NH, Hellsten U, Kawashima T, Kuo A, Mitros T, Salamov A, Carpenter ML (2008). The Trichoplax genome and the nature of placozoans. Nature.

[6] Srivastava M, Simakov O, Chapman J, Fahey B, Gauthier ME, Mitros T, Richards GS, Conaco C, Dacre M, Hellsten U (2010). The Amphimedon queenslandica genome and the evolution of animal complexity. Nature.

[7] Dambacher S, Hahn M, Schotta G (2010). Epigenetic regulation of development by histone lysine methylation. Heredity (Edinb).

[8] Chen T, Dent SY (2014). Chromatin modifiers and remodellers: regulators of cellular differentiation. Nat Rev Genet.

[9] Penalosa-Ruiz G, Bright AR, Mulder KW, Veenstra GJC (2019). The interplay of chromatin and transcription factors during cell fate transitions in development and reprogramming. Biochim Biophys Acta Gene Regul Mech.

[10] Grau-Bove X, Navarrete C, Chiva C, Pribasnig T, Anto M, Torruella G, Galindo LJ, Lang BF, Moreira D, Lopez-Garcia P (2022). A phylogenetic and proteomic reconstruction of eukaryotic chromatin evolution. Nat Ecol Evol.

[11] Andres ME, Burger C, Peral-Rubio MJ, Battaglioli E, Anderson ME, Grimes J, Dallman J, Ballas N, Mandel G (1999). CoREST: a functional corepressor required for regulation of neural-specific gene expression. Proc Natl Acad Sci U S A.

[12] Ballas N, Battaglioli E, Atouf F, Andres ME, Chenoweth J, Anderson ME, Burger C, Moniwa M, Davie JR, Bowers WJ (2001). Regulation of neuronal traits by a novel transcriptional complex. Neuron.

[13] Ballas N, Grunseich C, Lu DD, Speh JC, Mandel G (2005). REST and its corepressors mediate plasticity of neuronal gene chromatin throughout neurogenesis. Cell.

[14] Hakimi MA, Dong Y, Lane WS, Speicher DW, Shiekhattar R (2003). A candidate X-linked mental retardation gene is a component of a new family of histone deacetylase-containing complexes. J Biol Chem.

[15] Hakimi MA, Bochar DA, Chenoweth J, Lane WS, Mandel G, Shiekhattar R (2002). A core-BRAF35 complex containing histone deacetylase mediates repression of neuronal-specific genes. Proc Natl Acad Sci U S A.

[16] You A, Tong JK, Grozinger CM, Schreiber SL (2001). CoREST is an integral component of the CoREST- human histone deacetylase complex. Proc Natl Acad Sci U S A.

[17] Shi Y, Sawada J, Sui G, el Affar B, Whetstine JR, Lan F, Ogawa H, Luke MP, Nakatani Y, Shi Y (2003). Coordinated histone modifications mediated by a CtBP co-repressor complex. Nature.

[18] Mulligan P, Yang F, Di Stefano L, Ji JY, Ouyang J, Nishikawa JL, Toiber D, Kulkarni M, Wang Q, Najafi-Shoushtari SH (2011). A SIRT1-LSD1 corepressor complex regulates Notch target gene expression and development. Mol Cell.

[19] Shi Y, Lan F, Matson C, Mulligan P, Whetstine JR, Cole PA, Casero RA, Shi Y (2004). Histone demethylation mediated by the nuclear amine oxidase homolog LSD1. Cell.

[20] Yang M, Gocke CB, Luo X, Borek D, Tomchick DR, Machius M, Otwinowski Z, Yu H (2006). Structural basis for CoREST-dependent demethylation of nucleosomes by the human LSD1 histone demethylase. Mol Cell.

[21] Forneris F, Binda C, Dall'Aglio A, Fraaije MW, Battaglioli E, Mattevi A (2006). A highly specific mechanism of histone H3–K4 recognition by histone demethylase LSD1. J Biol Chem.

[22] Pilotto S, Speranzini V, Tortorici M, Durand D, Fish A, Valente S, Forneris F, Mai A, Sixma TK, Vachette P (2015). Interplay among nucleosomal DNA, histone tails, and corepressor CoREST underlies LSD1-mediated H3 demethylation. Proc Natl Acad Sci U S A.

[23] Song Y, Dagil L, Fairall L, Robertson N, Wu M, Ragan TJ, Savva CG, Saleh A, Morone N, Kunze MBA (2020). Mechanism of Crosstalk between the LSD1 Demethylase and HDAC1 Deacetylase in the CoREST Complex. Cell Rep.

[24] Shi YJ, Matson C, Lan F, Iwase S, Baba T, Shi Y (2005). Regulation of LSD1 histone demethylase activity by its associated factors. Mol Cell.

[25] Lee MG, Wynder C, Cooch N, Shiekhattar R (2005). An essential role for CoREST in nucleosomal histone 3 lysine 4 demethylation. Nature.

[26] Chen Y, Yang Y, Wang F, Wan K, Yamane K, Zhang Y, Lei M (2006). Crystal structure of human histone lysine-specific demethylase 1 (LSD1). Proc Natl Acad Sci U S A.

[27] Stavropoulos P, Blobel G, Hoelz A (2006). Crystal structure and mechanism of human lysine-specific demethylase-1. Nat Struct Mol Biol.

[28] Forneris F, Binda C, Adamo A, Battaglioli E, Mattevi A (2007). Structural basis of LSD1-CoREST selectivity in histone H3 recognition. J Biol Chem.

[29] Macinkovic I, Theofel I, Hundertmark T, Kovac K, Awe S, Lenz J, Forne I, Lamp B, Nist A, Imhof A (2019). Distinct CoREST complexes act in a cell-type-specific manner. Nucleic Acids Res.

[30] Kim HM, Beese-Sims SE, Colaiacovo MP (2018). Fanconi Anemia FANCM/FNCM-1 and FANCD2/FCD-2 Are Required for Maintaining Histone Methylation Levels and Interact with the Histone Demethylase LSD1/SPR-5 in Caenorhabditis elegans. Genetics.

[31] Eimer S, Lakowski B, Donhauser R, Baumeister R (2002). Loss of spr-5 bypasses the requirement for the C.elegans presenilin sel-12 by derepressing hop-1. EMBO J.

[32] Fuentes P, Canovas J, Berndt FA, Noctor SC, Kukuljan M (2012). CoREST/LSD1 control the development of pyramidal cortical neurons. Cereb Cortex.

[33] Lopez CI, Saud KE, Aguilar R, Berndt FA, Canovas J, Montecino M, Kukuljan M (2016). The chromatin modifying complex CoREST/LSD1 negatively regulates notch pathway during cerebral cortex development. Dev Neurobiol.

[34] Saijo K, Winner B, Carson CT, Collier JG, Boyer L, Rosenfeld MG, Gage FH, Glass CK (2009). A Nurr1/CoREST pathway in microglia and astrocytes protects dopaminergic neurons from inflammation-induced death. Cell.

[35] Upadhyay G, Chowdhury AH, Vaidyanathan B, Kim D, Saleque S (2014). Antagonistic actions of Rcor proteins regulate LSD1 activity and cellular differentiation. Proc Natl Acad Sci U S A.

[36] Monaghan CE, Nechiporuk T, Jeng S, McWeeney SK, Wang J, Rosenfeld MG, Mandel G (2017). REST corepressors RCOR1 and RCOR2 and the repressor INSM1 regulate the proliferation-differentiation balance in the developing brain. Proc Natl Acad Sci U S A.

[37] Wang Y, Wu Q, Yang P, Wang C, Liu J, Ding W, Liu W, Bai Y, Yang Y, Wang H (2016). LSD1 co-repressor Rcor2 orchestrates neurogenesis in the developing mouse brain. Nat Commun.

[38] Boxer LD, Barajas B, Tao S, Zhang J, Khavari PA (2014). ZNF750 interacts with KLF4 and RCOR1, KDM1A, and CTBP1/2 chromatin regulators to repress epidermal progenitor genes and induce differentiation genes. Genes Dev.

[39] Xiong Y, Wang L, Di Giorgio E, Akimova T, Beier UH, Han R, Trevisanut M, Kalin JH, Cole PA, Hancock WW (2020). Inhibiting the coregulator CoREST impairs Foxp3+ Treg function and promotes antitumor immunity. J Clin Invest.

[40] Saleque S, Kim J, Rooke HM, Orkin SH (2007). Epigenetic regulation of hematopoietic differentiation by Gfi-1 and Gfi-1b is mediated by the cofactors CoREST and LSD1. Mol Cell.

[41] Lee MC, Spradling AC (2014). The progenitor state is maintained by lysine-specific demethylase 1-mediated epigenetic plasticity during Drosophila follicle cell development. Genes Dev.

[42] Di Stefano L, Ji JY, Moon NS, Herr A, Dyson N (2007). Mutation of Drosophila Lsd1 disrupts H3–K4 methylation, resulting in tissue-specific defects during development. Curr Biol.

[43] Carpenter BS, Scott A, Goldin R, Chavez SR, Myrick DA, Curlee M, Schmeichel K, Katz DJ. CoREST has a conserved role in facilitating SPR-5/LSD1 maternal reprogramming of histone methylation. bioRxiv. 2021. 10.1101/2021.05.17.444472.

[44] Katz DJ, Edwards TM, Reinke V, Kelly WG (2009). A C. elegans LSD1 demethylase contributes to germline immortality by reprogramming epigenetic memory. Cell.

[45] Wasson JA, Simon AK, Myrick DA, Wolf G, Driscoll S, Pfaff SL, Macfarlan TS, Katz DJ (2016). Maternally provided LSD1/KDM1A enables the maternal-to-zygotic transition and prevents defects that manifest postnatally. Elife.

[46] Layden MJ, Rentzsch F, Rottinger E (2016). The rise of the starlet sea anemone Nematostella vectensis as a model system to investigate development and regeneration. Wiley Interdiscip Rev Dev Biol.

[47] Rentzsch F, Juliano C, Galliot B (2019). Modern genomic tools reveal the structural and cellular diversity of cnidarian nervous systems. Curr Opin Neurobiol.

[48] Hand C, Uhlinger KR (1992). The Culture, Sexual and Asexual Reproduction, and Growth of the Sea Anemone Nematostella vectensis. Biol Bull.

[49] Magie CR, Daly M, Martindale MQ (2007). Gastrulation in the cnidarian Nematostella vectensis occurs via invagination not ingression. Dev Biol.

[50] Fritzenwanker JH, Genikhovich G, Kraus Y, Technau U (2007). Early development and axis specification in the sea anemone Nematostella vectensis. Dev Biol.

[51] Gahan JM, Kouzel IU, Jansen KO, Burkhardt P, Rentzsch F (2022). Histone demethylase Lsd1 is required for the differentiation of neural cells in Nematostella vectensis. Nat Commun.

[52] Zhou X, Ma H (2008). Evolutionary history of histone demethylase families: distinct evolutionary patterns suggest functional divergence. BMC Evol Biol.

[53] Gregoretti IV, Lee YM, Goodson HV (2004). Molecular evolution of the histone deacetylase family: functional implications of phylogenetic analysis. J Mol Biol.

[54] Milazzo G, Mercatelli D, Di Muzio G, Triboli L, De Rosa P, Perini G, Giorgi FM (2020). Histone Deacetylases (HDACs): evolution, specificity, role in transcriptional complexes, and pharmacological actionability. Genes (Basel).

[55] Planques A, Kerner P, Ferry L, Grunau C, Gazave E, Vervoort M (2021). DNA methylation atlas and machinery in the developing and regenerating annelid Platynereis dumerilii. BMC Biol.

[56] Olivares M, Merello G, Verbel Vergara D, Gonzalez M, Andrés M, Opazo J (2021). Evolution of Lysine-Specific Demethylase 1 and REST Corepressor Gene Families and Their Molecular Interaction.

[57] Lan F, Collins RE, De Cegli R, Alpatov R, Horton JR, Shi X, Gozani O, Cheng X, Shi Y (2007). Recognition of unmethylated histone H3 lysine 4 links BHC80 to LSD1-mediated gene repression. Nature.

[58] Rivero S, Ceballos-Chavez M, Bhattacharya SS, Reyes JC (2015). HMG20A is required for SNAI1-mediated epithelial to mesenchymal transition. Oncogene.

[59] Iwase S, Januma A, Miyamoto K, Shono N, Honda A, Yanagisawa J, Baba T (2004). Characterization of BHC80 in BRAF-HDAC complex, involved in neuron-specific gene repression. Biochem Biophys Res Commun.

[60] Sunagar K, Columbus-Shenkar YY, Fridrich A, Gutkovich N, Aharoni R, Moran Y (2018). Cell type-specific expression profiling unravels the development and evolution of stinging cells in sea anemone. BMC Biol.

[61] Busengdal H, Rentzsch F (2017). Unipotent progenitors contribute to the generation of sensory cell types in the nervous system of the cnidarian Nematostella vectensis. Dev Biol.

[62] Nakanishi N, Renfer E, Technau U, Rentzsch F (2012). Nervous systems of the sea anemone Nematostella vectensis are generated by ectoderm and endoderm and shaped by distinct mechanisms. Development.

[63] Beckmann A, Ozbek S (2012). The nematocyst: a molecular map of the cnidarian stinging organelle. Int J Dev Biol.

[64] Marlow HQ, Srivastava M, Matus DQ, Rokhsar D, Martindale MQ (2009). Anatomy and development of the nervous system of Nematostella vectensis, an anthozoan cnidarian. Dev Neurobiol.

[65] Szczepanek S, Cikala M, David CN (2002). Poly-gamma-glutamate synthesis during formation of nematocyst capsules in Hydra. J Cell Sci.

[66] Zenkert C, Takahashi T, Diesner MO, Ozbek S (2011). Morphological and molecular analysis of the Nematostella vectensis cnidom. PLoS One.

[67] Tourniere O, Dolan D, Richards GS, Sunagar K, Columbus-Shenkar YY, Moran Y, Rentzsch F (2020). NvPOU4/Brain3 functions as a terminal selector gene in the nervous system of the cnidarian nematostella vectensis. Cell Rep.

[68] Dallman JE, Allopenna J, Bassett A, Travers A, Mandel G (2004). A conserved role but different partners for the transcriptional corepressor CoREST in fly and mammalian nervous system formation. J Neurosci.

[69] Laurent B, Ruitu L, Murn J, Hempel K, Ferrao R, Xiang Y, Liu S, Garcia BA, Wu H, Wu F (2015). A specific LSD1/KDM1A isoform regulates neuronal differentiation through H3K9 demethylation. Mol Cell.

[70] Zibetti C, Adamo A, Binda C, Forneris F, Toffolo E, Verpelli C, Ginelli E, Mattevi A, Sala C, Battaglioli E (2010). Alternative splicing of the histone demethylase LSD1/KDM1 contributes to the modulation of neurite morphogenesis in the mammalian nervous system. J Neurosci.

[71] Toffolo E, Rusconi F, Paganini L, Tortorici M, Pilotto S, Heise C, Verpelli C, Tedeschi G, Maffioli E, Sala C (2014). Phosphorylation of neuronal Lysine-Specific Demethylase 1LSD1/KDM1A impairs transcriptional repression by regulating interaction with CoREST and histone deacetylases HDAC1/2. J Neurochem.

[72] Rusconi F, Grillo B, Toffolo E, Mattevi A, Battaglioli E (2017). NeuroLSD1: splicing-generated epigenetic enhancer of neuroplasticity. Trends Neurosci.

[73] Gahan JM, Rentzsch F, Schnitzler CE (2020). The genetic basis for PRC1 complex diversity emerged early in animal evolution. Proc Natl Acad Sci.

[74] Fritzenwanker JH, Technau U (2002). Induction of gametogenesis in the basal cnidarian Nematostella vectensis(Anthozoa). Dev Genes Evol.

[75] Richter DJ, Fozouni P, Eisen MB, King N (2018). Gene family innovation, conservation and loss on the animal stem lineage. eLife.

[76] Grau-Bové X, Torruella G, Donachie S, Suga H, Leonard G, Richards TA, Ruiz-Trillo I (2017). Dynamics of genomic innovation in the unicellular ancestry of animals. eLife.

[77] Torruella G, de Mendoza A, Grau-Bové X, Antó M, Chaplin MA, del Campo J, Eme L, Pérez-Cordón G, Whipps CM, Nichols KM (2015). Phylogenomics reveals convergent evolution of lifestyles in close relatives of animals and fungi. Curr Biol.

[78] Katoh K, Misawa K, Kuma K, Miyata T (2002). MAFFT: a novel method for rapid multiple sequence alignment based on fast Fourier transform. Nucleic Acids Res.

[79] Kumar S, Stecher G, Tamura K (2016). MEGA7: molecular evolutionary genetics analysis version 7.0 for Bigger datasets. Mol Biol Evol.

[80] Perez-Riverol Y, Bai J, Bandla C, García-Seisdedos D, Hewapathirana S, Kamatchinathan S, Kundu Deepti J, Prakash A, Frericks-Zipper A, Eisenacher M (2021). The PRIDE database resources in 2022: a hub for mass spectrometry-based proteomics evidences. Nucleic Acids Res.

[81] Cox J, Mann M (2008). MaxQuant enables high peptide identification rates, individualized p.p.b.-range mass accuracies and proteome-wide protein quantification. Nat Biotechnol.

[82] Tyanova S, Temu T, Sinitcyn P, Carlson A, Hein MY, Geiger T, Mann M, Cox J (2016). The Perseus computational platform for comprehensive analysis of (prote)omics data. Nat Methods.

[83] Ikmi A, McKinney SA, Delventhal KM, Gibson MC (2014). TALEN and CRISPR/Cas9-mediated genome editing in the early-branching metazoan Nematostella vectensis. Nat Commun.

[84] Kraus Y, Aman A, Technau U, Genikhovich G (2016). Pre-bilaterian origin of the blastoporal axial organizer. Nat Commun.

[85] Renfer E, Amon-Hassenzahl A, Steinmetz PR, Technau U (2010). A muscle-specific transgenic reporter line of the sea anemone, Nematostella vectensis. Proc Natl Acad Sci U S A.

[86] Sievers F, Wilm A, Dineen D, Gibson TJ, Karplus K, Li W, Lopez R, McWilliam H, Remmert M, Soding J (2011). Fast, scalable generation of high-quality protein multiple sequence alignments using Clustal Omega. Mol Syst Biol.

