# Supplementary material for: A developmental role for the chromatin-regulating CoREST complex in the cnidarian Nematostella vectensis
Source: BMC Biol. 2022 Aug 23;20:184. doi: 10.1186/s12915-022-01385-1 (PMC9400249; doi:10.1186/s12915-022-01385-1)
Supplement: Supplementary file 1 — Additional file 1: Table S1-4. Table S1. Search for COREST-like in non-metazoan and metazoan genomes and transcriptomes. Table S2. Data from individual replicates of LC-MS experiments. Table S3. List of primers used in this study. Table S4. List of antibodies and dilutions. [file 12915_2022_1385_MOESM1_ESM.pdf]

Table S1. Search for COREST-like in non-metazoan and metazoan genomes and transcriptomes.

Clade	Species	ELM2-SANT	ELM2-SANT-SANT=CoREST	Blasted data	Source
Others (exclude opistokonta	All species	plant sequences : contamination	plant sequences : contamination	NCBI protein	NCBI
fungi	All species	present	0	NCBI protein	NCBI
Filasterea	*Capsaspora owczarzaki*	0	0	Genome	Ensembl Protists
Ichthyosporea	*Aboeforma whisleri*	2	0	Transcriptome	Torruella et al. 2015
Ichthyosporea	*Amoebidium parasiticum*	3	0	Transcriptome	Torruella et al. 2015
Ichthyosporea	*Chromosphaera perkinsii*	1	0	Transcriptome	Grau-bové et al. 2017
Ichthyosporea	*Ichthyophonus hoferi*	3	0	Transcriptome	Torruella et al. 2015
Ichthyosporea	*Pirum gemmata*	1	0	Transcriptome	Torruella et al. 2015
Ichthyosporea	*Sphaeroforma arctica*	1	0	Genome	Ensembl Protists
Ichthyosporea	*Sphaerothecum destruens*	1	0	Transcriptome	Torruella et al. 2015
Terestosporea	*Corallochytrium limacisporum*	0	0	Transcriptome	Torruella et al. 2015
Choanoflagellata	*Acanthoeca spectabilis*	0	0	Transcriptome	Richter et al. 2018
Choanoflagellata	*Choanoeca perplexa*	1	0	Transcriptome	Richter et al. 2018
Choanoflagellata	*Codosiga hollandica*	1	0	Transcriptome	Richter et al. 2018
Choanoflagellata	***Diaphanoeca grandis***	3	1	Transcriptome	Richter et al. 2018
Choanoflagellata	***Didymoeca costata***	1	1	Transcriptome	Richter et al. 2018
Choanoflagellata	*Hartaetosiga balthica*	0	0	Transcriptome	Richter et al. 2018
Choanoflagellata	*Hartaetosiga gracilis*	0	0	Transcriptome	Richter et al. 2018
Choanoflagellata	***Helgoeca nana***	1	1	Transcriptome	Richter et al. 2018
Choanoflagellata	*Microstomoeca roanoka*	0	0	Transcriptome	Richter et al. 2018
Choanoflagellata	*Monosiga brevicolis MX1*	0	0	Genome	Ensembl Protists
Choanoflagellata	*Mylnosiga fluctuans*	1	0	Transcriptome	Richter et al. 2018
Choanoflagellata	***Salpingoeca dolichothecata***	2	1	Transcriptome	Richter et al. 2018
Choanoflagellata	*Salpingoeca helianthica*	1	0	Transcriptome	Richter et al. 2018
Choanoflagellata	*Salpingoeca infusionum*	0	0	Transcriptome	Richter et al. 2018
Choanoflagellata	*Salpingoeca kvevrii*	1	0	Transcriptome	Richter et al. 2018
Choanoflagellata	*Salpingoeca macrocollata*	1	0	Transcriptome	Richter et al. 2018
Choanoflagellata	***Salpingoeca punica***	1	**1**	Transcriptome	Richter et al. 2018
Choanoflagellata	*Salpingoeca rosetta*	0	0	Genome	Ensembl Protists
Choanoflagellata	*Salpingoeca urceolata*	0	0	Transcriptome	Richter et al. 2018
Choanoflagellata	*Savillea parva*	0	0	Transcriptome	Richter et al. 2018
Choanoflagellata	*Stephanoeca diplocostata.Australia*	0	0	Transcriptome	Richter et al. 2018
Choanoflagellata	*Stephanoeca diplocostata.France*	0	0	Transcriptome	Richter et al. 2018

Clade	Species	CoREST	Blasted data	Source
Ctenophora	*Pleurobrachia pileus*	1	Genome	neurobase.rc.ufl.edu
Ctenophora	*Mnemiopsis leidyi*	1	Genome	Ensembl Metazoa
Ctenophora	*Other transcriptomes*	present	Transcriptomes	neurobase.rc.ufl.edu
Demospongiae	*Amphimedon queenslandica*	1	Genome	NCBI nr
Demospongiae	*Ephydatia muelleri*	1	Transcriptome	compagen.org
Homoscleromorpha	*Oscarella carmela*	1	Genome	compagen.org
Placozoa	*Trichoplax adhaerens*	1	Genome	NCBI nr
Anthozoa	*Nematostella vectensis*	1	Genome	NCBI nr
Anthozoa	*Exaiptasia diaphana*	1	Genome	NCBI nr
Anthozoa	*Acropora millepora*	1	Genome	NCBI nr
Anthozoa	*Stylophora pistillata*	1	Genome	NCBI nr
Anthozoa	*Xenia sp.*	1	Genome	NCBI nr
Anthozoa	*Actinia tenebrosa*	1	Genome	NCBI nr
Anthozoa	*Dendronephthya gigantea*	1	Genome	NCBI nr
Anthozoa	*Pocillopora damicornis*	1	Genome	NCBI nr
Hydrozoa	*Hydra vulgaris*	1	Genome	research.nhgri.nih.gov/hydra
Hydrozoa	*Clytia hemisphaerica*	1	Genome	Ensembl Metazoa
Scyphozoa	*Pelagia noctiluca*	1	Transcriptome	Unpublished data
Scyphozoa	*Aurelia aurita*	1	Genome	marinegenomics.oist.jp
Cubozoa	*Morbakka virulenta*	1	Genome	marinegenomics.oist.jp
Acoela	*Praesagittifera naikaiensis*	2	Genome	marinegenomics.oist.jp
Priapulida	*Priapulus caudatus*	1	Transcriptome	NCBI nr
Nematoda	*Caenorhabditis elegans*	1	Genome	NCBI nr
Nematoda	*Brugia malayi*	1	Genome	NCBI nr
Nematoda	*Loa loa*	1	Genome	NCBI nr
Arthropoda	*Sarcoptes scabiei*	1	Genome	NCBI nr
Arthropoda	*Stegodyphus mimosarum*	1	Genome	NCBI nr
Arthropoda	*Ixodes scapularis*	1	Genome	NCBI nr
Arthropoda	*Tetranychus urticae*	1	Genome	NCBI nr
Brachiopoda	*Lingula anatina*	1	Genome	NCBI nr/ TSA
Bryozoa	*Bugula neritina*	3	Genome	NCBI nr
Phoronida	*Phoronis australis*	1	Transcriptome	marinegenomics.oist.jp
Nemertean	*Notospermus geniculatus*	2	Genome	marinegenomics.oist.jp
Annelida	*Capitella teleta*	1	Genome	Ensembl Metazoa
Annelida	*Helobdella robusta*	1	Genome	Ensembl Metazoa
Rotifera	*Adineta vaga*	1	Genome	Ensembl Metazoa
Mollusca	*Crassostrea gigas*	1	Genome	NCBI nr
Mollusca	*Haliotis rufescens*	1	Genome	NCBI nr
Mollusca	*Octopus bimaculoides*	1	Genome	NCBI nr
Echinodermata	*Strongylocentrotus purpuratus*	1	Genome	NCBI nr
Echinodermata	*Lytechinus variegatus*	1	Transcriptome	NCBI nr
Echinodermata	*Acanthaster planci*	1	Genome	NCBI nr
Hemichordata	*Saccoglossus kowalevskii*	1	Genome	NCBI nr
Hemichordata	*Ptychodera flava*	1	Genome	marinegenomics.oist.jp
Cephalochordata	*Branchiostoma floridae*	1	Genome	NCBI nr
Urochordata	*Ciona intestinalis*	1	Genome	NCBI nr/Aniseed
Urochordata	*Phallusia mammilata*	1	Genome	NCBI nr/Aniseed
Petromyzontiformes	*Petromyzon marinus*	1	Genome	NCBI nr
Hagfish	*Eptatretus burgeri*	1	Genome	Ensembl
Homo sapiens	Homo sapiens	3	Genome	NCBI nr
Xenopus tropicalis	Xenopus tropicalis	3	Genome	NCBI nr
Mus musculus	Mus musculus	3	Genome	NCBI nr
Danio rerio	Danio rerio	3	Genome	NCBI nr

Shark	Callorhinchus milii	3	Genome	NCBI nr

Table S2. Data from individual replicates of LC-MS experiments.

Name	UniProt ID	NVE annotation	Control 1	Control 2	Control 3	GFP 1	GFP 2	GFP 3	Median Control	Median GFP	Fold Change
GFP	P42212	NaN	0.51	0.79	NaN	9.77	9.64	9.47	0.65	9.64	8.99
NVPHD21A	A7RH46	NVE16417	NaN	-4.68	-4.00	6.19	5.61	6.66	-4.34	6.19	11.03
NvHMG20	A7S5L8	NVE23581	-2.12	-5.17	-0.23	6.77	5.60	7.55	-2.11	6.77	8.89
NvLsd1	A7S5A0	NVE23413	2.05	2.74	0.09	10.94	10.84	10.46	2.05	10.84	8.78
NvCoREST	A7SSF7	NVE11839	0.63	0.30	0.71	9.00	8.36	9.51	0.63	9.01	8.38
NvHDAC1/2	A7RFA3	NVE222	0.34	1.39	1.36	8.24	7.53	9.36	1.35	8.24	6.88

Table S3. List of primers used in this study.

Primer name	Sequence (5'-3')
NvCoREST-sgRNA1_Fwd (Used to generate NvCoREST Mutant 1)	TTCTAATACGACTCACTATAGGCGAGCCAACACGCA TCCCGTTTTAGAGCTAGA
NvCoREST-sgRNA2_Fwd (Used to generate NvCoREST Mutant 1)	TTCTAATACGACTCACTATAGGCAAGACACTGTGCA TTATGTTTTAGAGCTAGA
sgRNA_Rev (Genreic and used for both sgRNAs)	AAAAGCACCGACTCGGTGCCACTTTTTCAAGTTGAT AACGGACTAGCCTTATTTTAACTTGCTATTTCTAGCT CTAAAAC
NvCoREST_Mutant1_Sequence_Fwd	GAATGAAGGAACTGCGATGGAGACTAAG
NvCoREST_Mutant1_Sequence_Rev	CCATCCGACACCATCTCGGG
NvCoREST_Mutant2_Sequence_Fwd	GACGCTTTCCACTTTATGCTTTCCC
NvCoREST_Mutant2_Sequence_Rev	GCTTACATTTACTATCTGTCATATTCTTGGTAGG
NvCoREST_Full-Lenght_Fwd	ATGGCTTCTAGCGGCCG
NvCoREST_Full-Lenght_Rev (Used for cloning the gene and RT-PCR)	TCAAGGTGCCATGGTCC
NvCoREST_NoSTOP_Rev (Used to amplify NvCoREST to clone in frame with mCherry	AGGTGCCATGGTCCTACCAAG
NvPOU4_Vector_Fwd	CACAGGACCTTGGTAGGACCATGGCACCTGCGG GCGGCGGCGGCAGC
NvPOU4_Vector_Rev	GCCACGGTTCCTCGGCCGCTAGAAGCCATCGTG GAGCACTCAGCACCAAC

Table S4 List of antibodies and dilutions

Name	Company	Catalogue number	Concentration (IF)	Concentration (Western)
Rabbit anti-DsRed	Clontech	632496	1:100	
Mouse anti-mCherry	Clontech	632543	1:100	
Chicken anti-GFP	Kerafast	EMU101	1:100	
Rabbit anti-GFP	Abcam	Ab290		1:20,000
Rabbit anti-NvCoREST	Custom	n/a	1:100	1:10,000
Goat anti-rabbit Alexa 488	Life Technologies	A11008	1:250	
Goat anti-rabbit Alexa 568	Life Technologies	A11011	1:250	
Goat anti-mouse Alexa 488	Life Technologies	A11001	1:250	
Goat anti-mouse Alexa 568	Life Technologies	A11004	1:250	
Goat anti-chicken Alexa 633	Life Technologies	A21103	1:250	
Goat anti-chicken Alexa 488	Life Technologies	A11039	1:250	
Goat Anti-Rabbit (HRP)	Abcam	Ab97051		1:10,000
