# Supplementary figures and images for: A developmental role for the chromatin-regulating CoREST complex in the cnidarian Nematostella vectensis
Source: BMC Biol. 2022 Aug 23;20:184. doi: 10.1186/s12915-022-01385-1 (PMC9400249; doi:10.1186/s12915-022-01385-1)

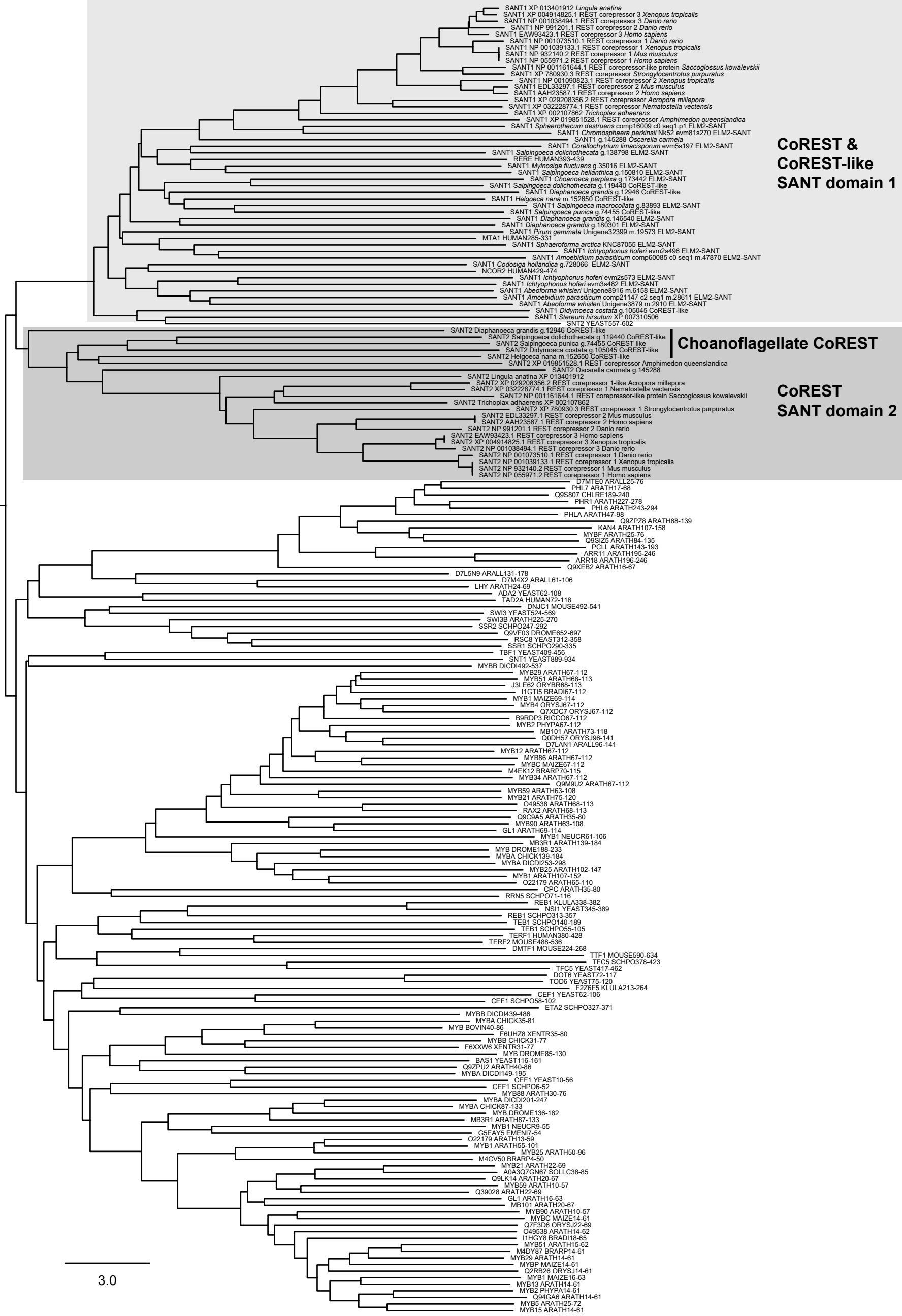

Supplement: Supplementary file 2 — Additional file 2: Fig S1. Neighbor-joining phylogeny of the SANT domain, including a selection of SANT domain sequences from various protein families, as well as metazoan and non-metazoan CoREST-like sequences. [file 12915_2022_1385_MOESM2_ESM.pdf]

CoREST & CoREST-like

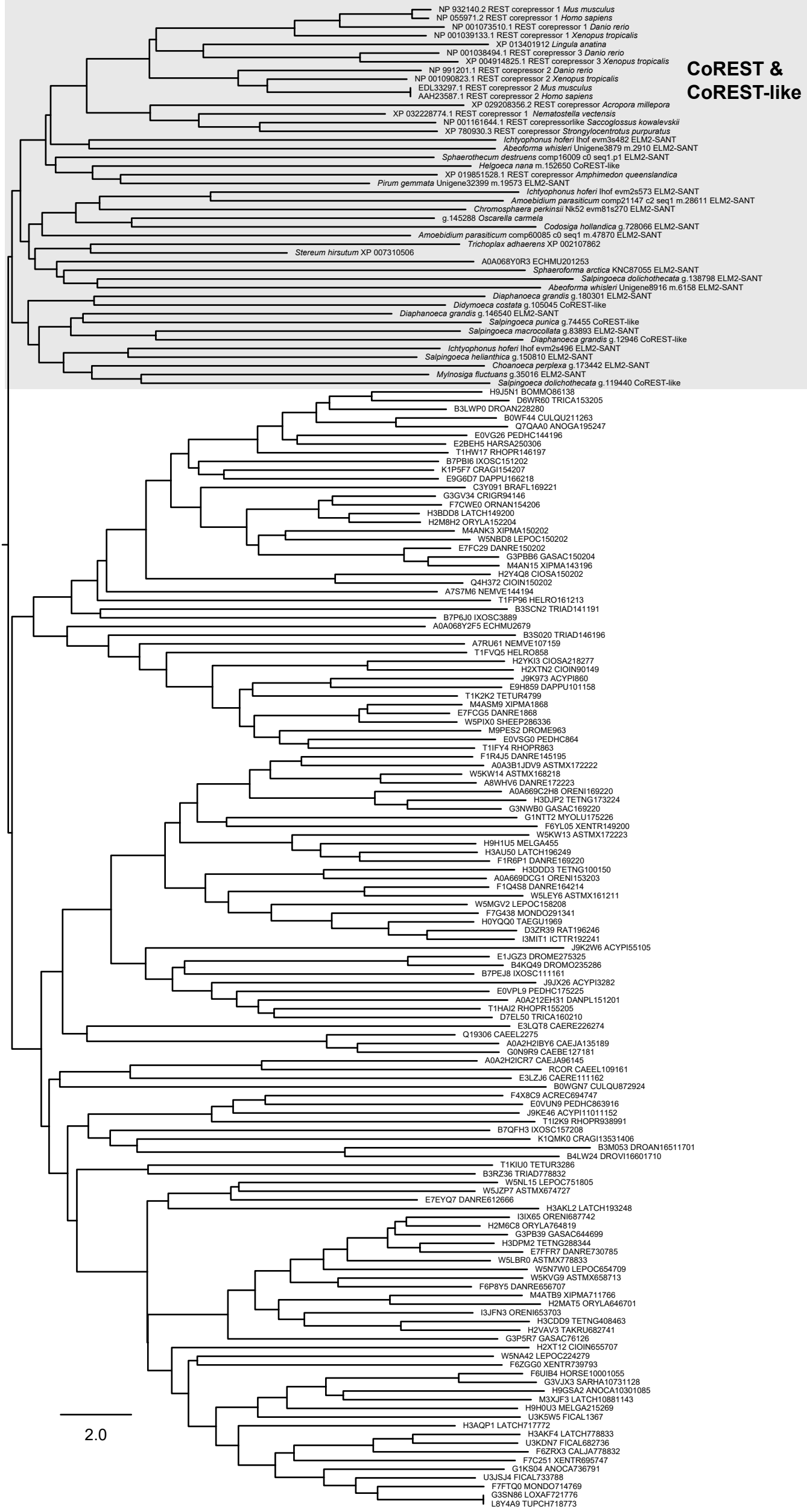

Supplement: Supplementary file 3 — Additional file 3: Fig S2. Neighbor-joining phylogeny of the ELM2 domain, including a selection of ELM2 domain sequences from various protein families, as well as metazoan and non-metazoan CoREST-like sequences. [file 12915_2022_1385_MOESM3_ESM.pdf]

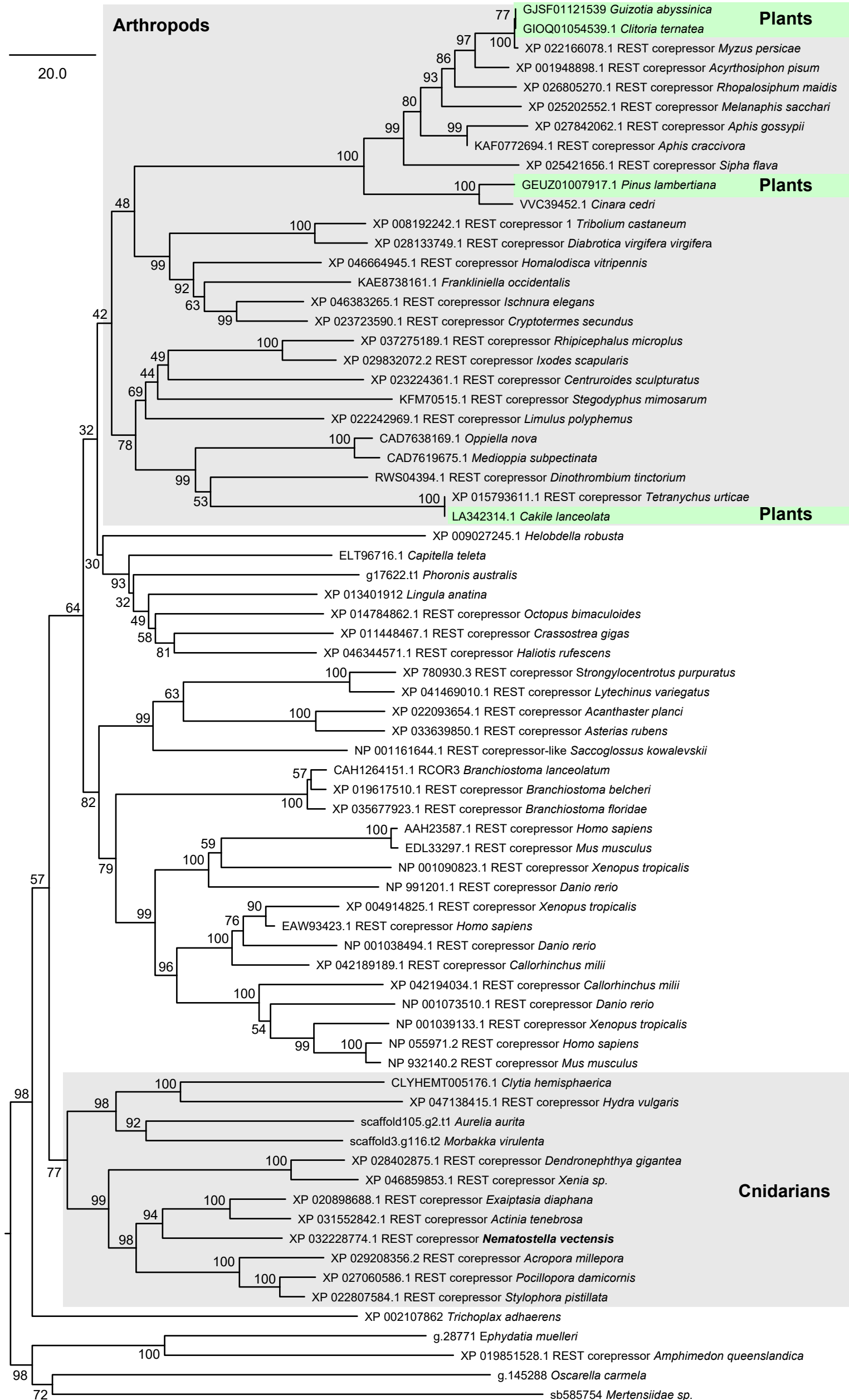

Supplement: Supplementary file 4 — Additional file 4: Fig S3. Neighbor-joining phylogeny of metazoan CoREST full protein. Bootstrap values are indicated next to the nodes. The tree is rooted with poriferan and ctenophore sequences. The distance scale represents the percentage of genetic or nucleotide variation between the sequences. The 4 “Plant CoREST-like” sequences retrieved from the NCBI TSA database group are likely arthropod contaminations as they cluster with insect and acharian CoREST sequences. [file 12915_2022_1385_MOESM4_ESM.pdf]

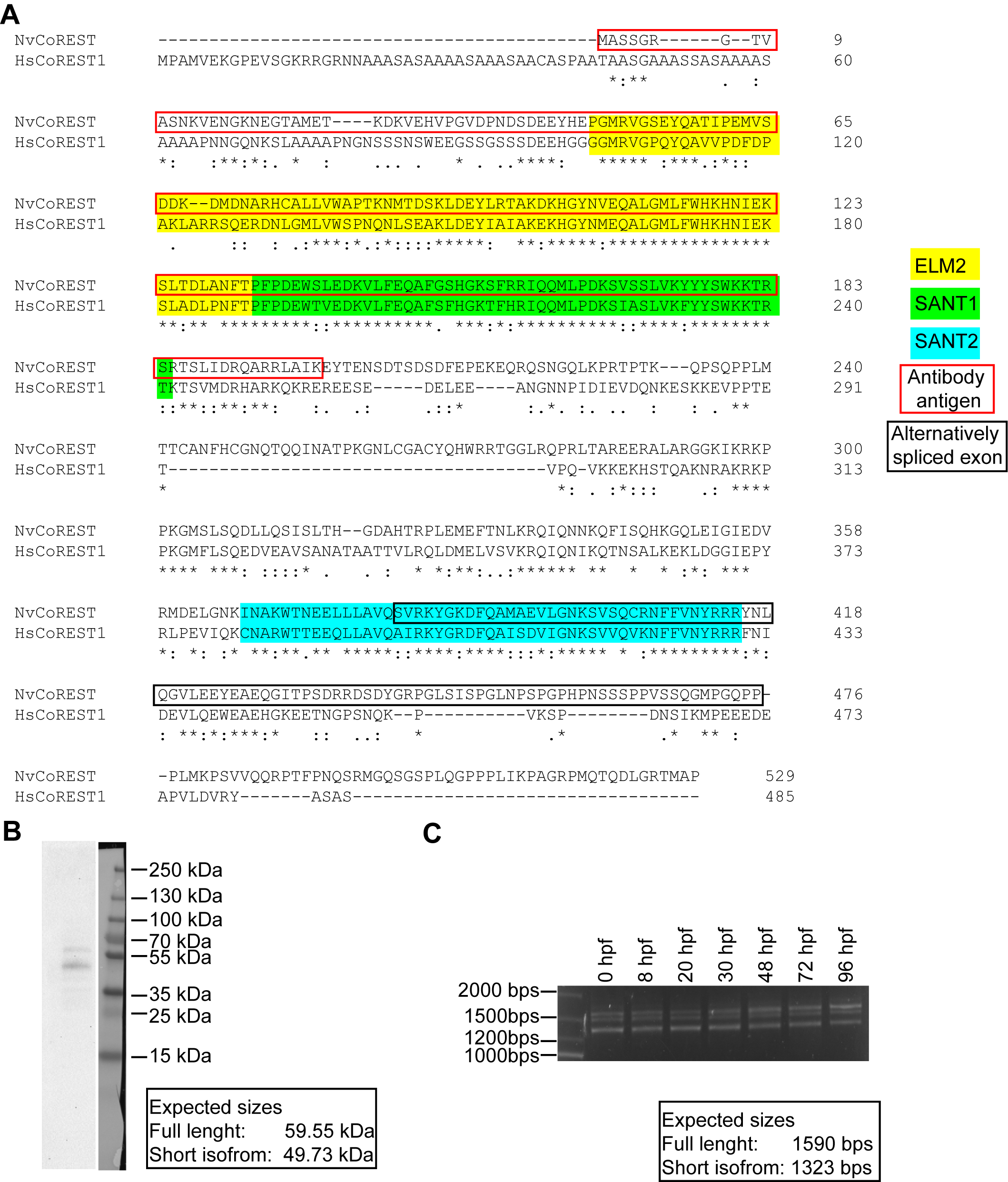

Supplement: Supplementary file 6 — Additional file 6: Fig S4. NvCoREST isoforms and antibody validation. (A) Alignment of full length Nematostella CoREST with human CoREST1 (UniProt: Q9UKL0). Alignment was performed using Clustal Omega [86]. Conserved domains are highlighted with coloured boxes; ELM2 in yellow, SANT1 in green and SANT2 in cyan. The portion of the protein used to generate the NvCoREST antibody is outlined with a red box. The alternatively spliced exon is outlined with a black box. (B) Western blot using anti-NvCoREST antibody showing two bands corresponding in size to the expected sizes of the full length and short isoform of NvCoREST, shown in the box at the bottom. Protein was extracted at planula stage. (C) PCR analysis using primers to amplify full length NvCoREST from cDNA from different developmental stages. The stage from which the cDNA was generated is shown on top measured in hours post fertilization (hpf). The expected sizes of full length and the short isoform of NvCoREST are shown in a box. Three bands are present; the highest and lowest correspond to the full length and short isoform of NvCoREST, respectively, and were successfully closed and sequenced. The middle band was never cloned and is presumably a PCR artifact, likely due to hybridization between the full length and short isoforms. Western blot and PCR analysis were carried out two times, independently with the same results. [file 12915_2022_1385_MOESM6_ESM.tif]

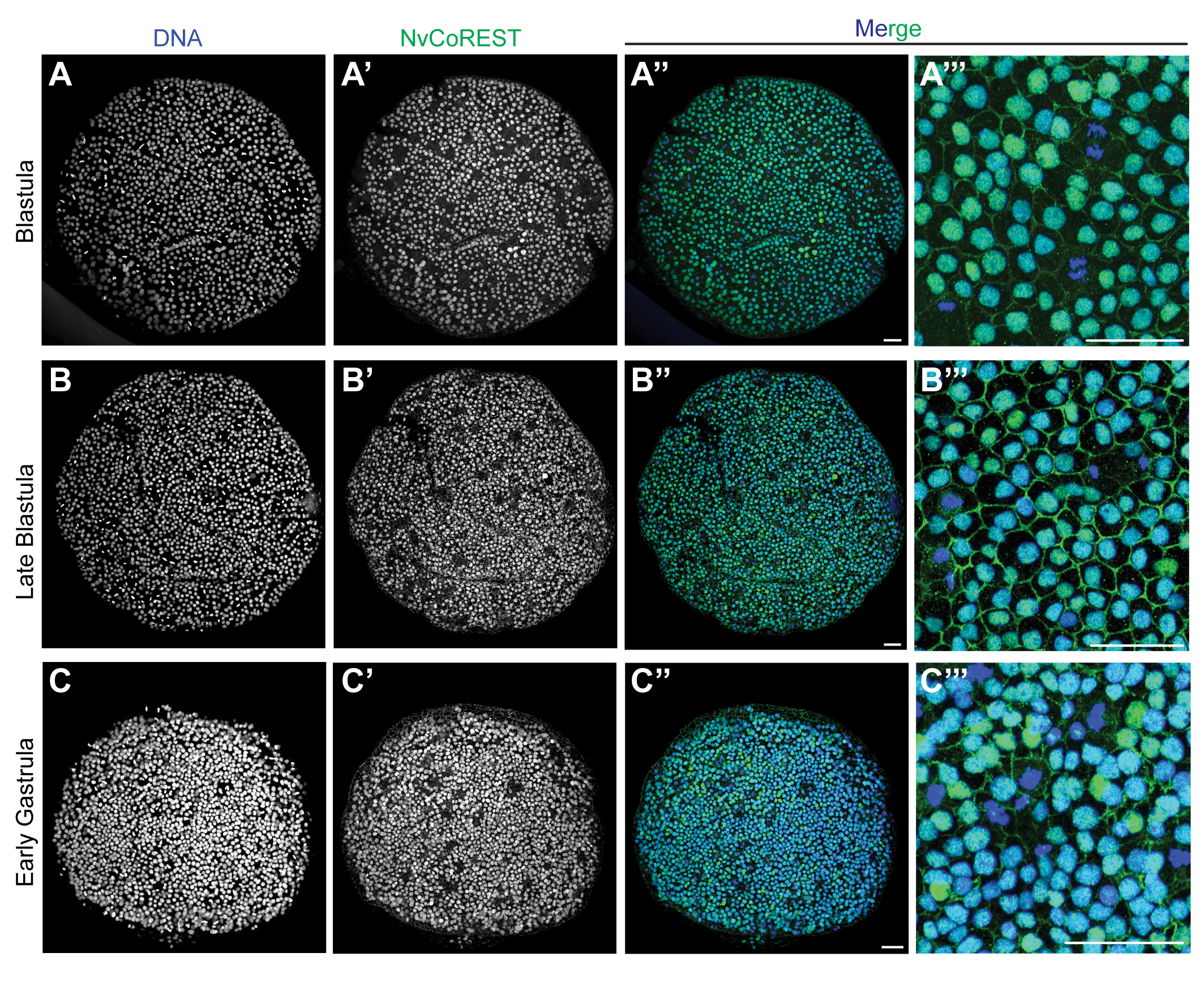

Supplement: Supplementary file 7 — Additional file 7: Fig S5. The heterogeneity in NvCoREST levels appears over developmental time. (A to C) Confocal images of immunofluorescence staining performed on early embryos. Stages used are indicated to the left of the images. (A’’’ to C’’’) show close ups. Staining’s were performed two times independently with a minimum of 10 embryos imaged per replicate with the same results. Scale bars: 20 µm. [file 12915_2022_1385_MOESM7_ESM.tif]

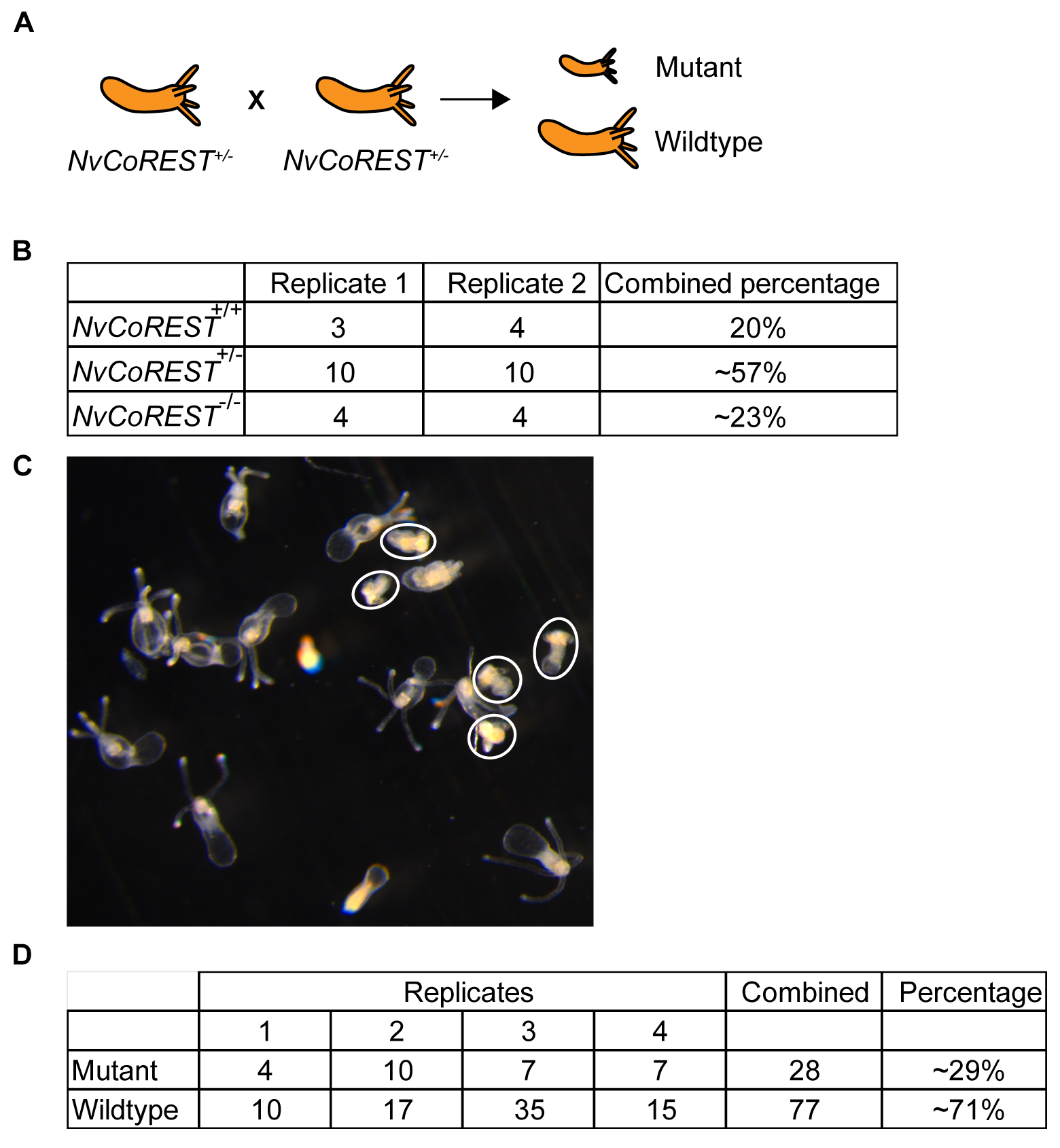

Supplement: Supplementary file 8 — Additional file 8: Fig S6. Additional data on NvCoREST mutants. (A) Schematic representation of the crosses used when analyzing NvCoREST mutants. (B) Genotyping results on larva derived from NvCoREST mutant 1 in-crosses at planula larva stage. The planulae showed no visible phenotype and were selected at random and genotyped by PCR and sequencing. (C) Brightfield image of live primary polyps derived from an in-cross of heterozygous NvCoREST mutant 1 animals. The animals displaying the mutant phenotype are highlighted with a white circle. (D) Data showing the number of animals with mutant or wildtype phenotype from 4 independent replicates. For each experiment 20-30 eggs were selected and grown to primary polyp stage and then those surviving were separated into either wildtype or mutant categories and counted. [file 12915_2022_1385_MOESM8_ESM.tif]

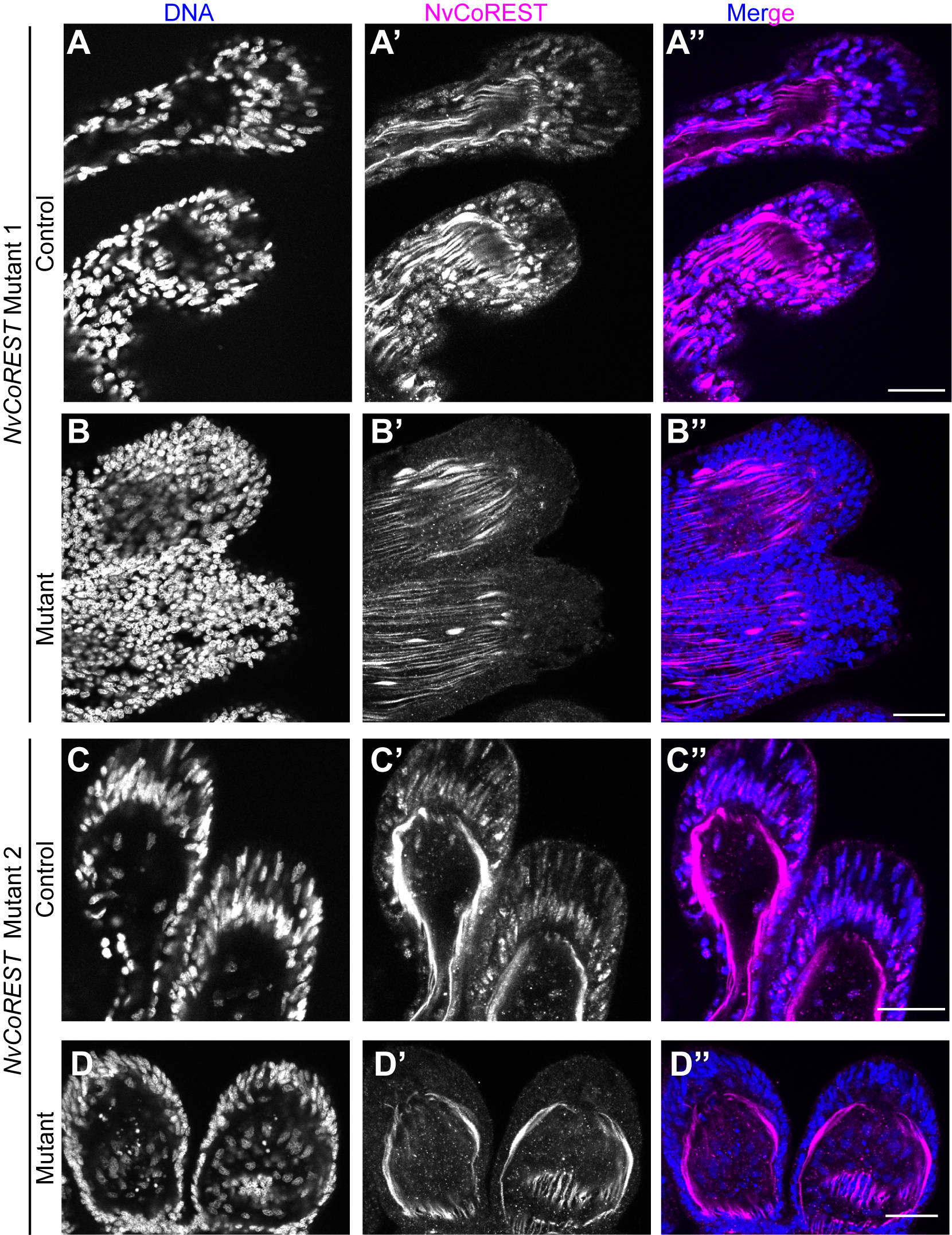

Supplement: Supplementary file 9 — Additional file 9: Fig S7. NvCoREST staining is absent in both NvCoREST mutant lines. (A to D) Confocal images of immunofluorescence staining on mutant and control primary polyps from NvCoREST Mutant 1 or 2 lines stained for NvCoREST (magenta) and DNA (blue). Mutant line and genotype are shown to the left. Ubiquitous nuclear NvCoREST staining can be seen in control but is absent in mutant animals while non-specific staining of actin filaments can be seen in both. Stainings were performed two times independently with a minimum of 10 embryos imaged per genotype, per replicate with the same results. Scale bars: 20 µm. [file 12915_2022_1385_MOESM9_ESM.tif]

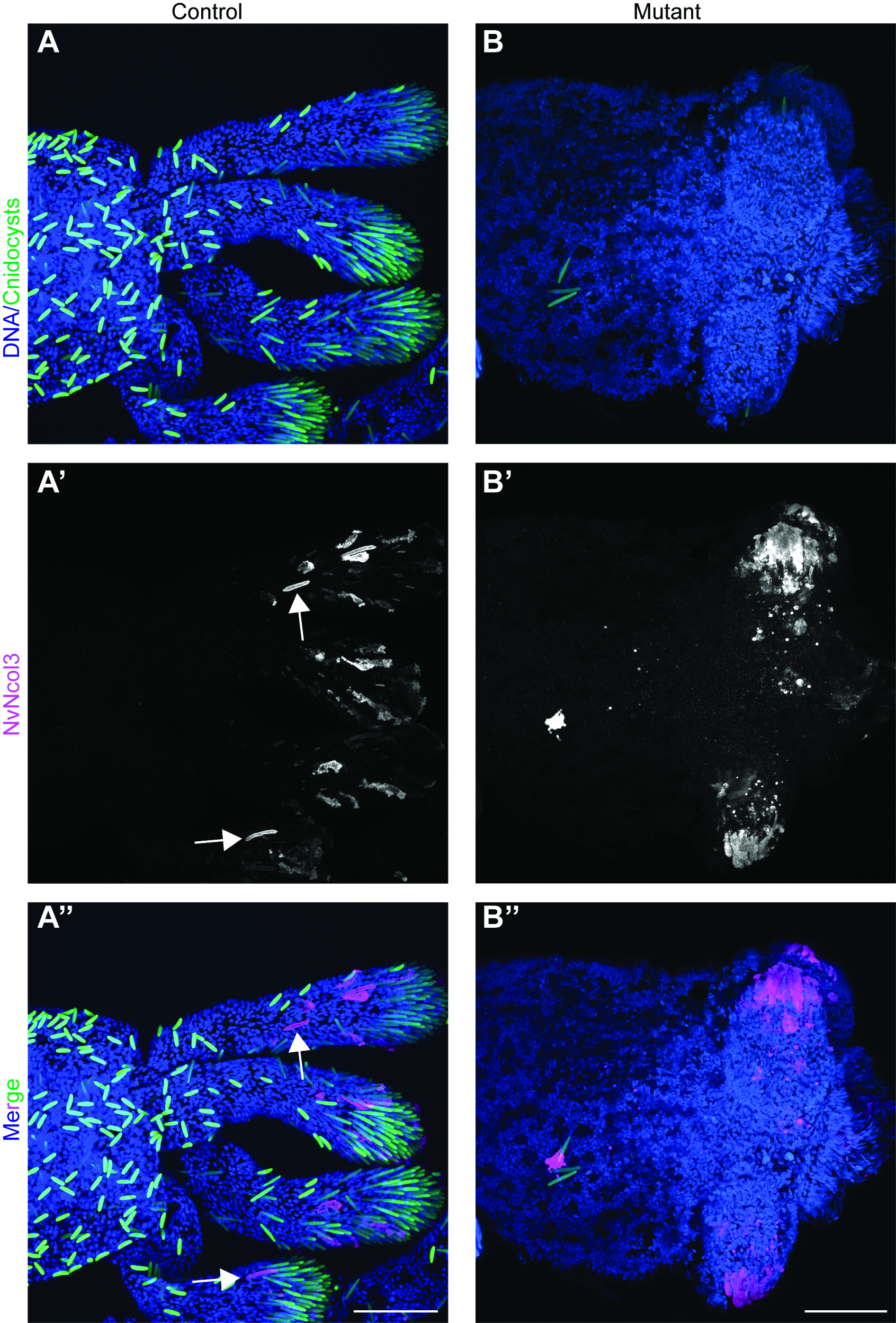

Supplement: Supplementary file 10 — Additional file 10: Fig S8. NvCoREST mutants still express NvNcol3. (A and B) Confocal images of immunofluorescence staining on NvCoREST Mutant 1 primary polyps showing NvNcol3 in Magenta, DNA in blue and cnidocysts in Green. Arrows in A’ and A’’ indicate developing cnidocysts with normal morphology. The experiment was performed twice, independently and a minimum of 10 animals per genotype, per replicate were analyzed and showed the same result. Scale bars: 50 µm. [file 12915_2022_1385_MOESM10_ESM.tif]

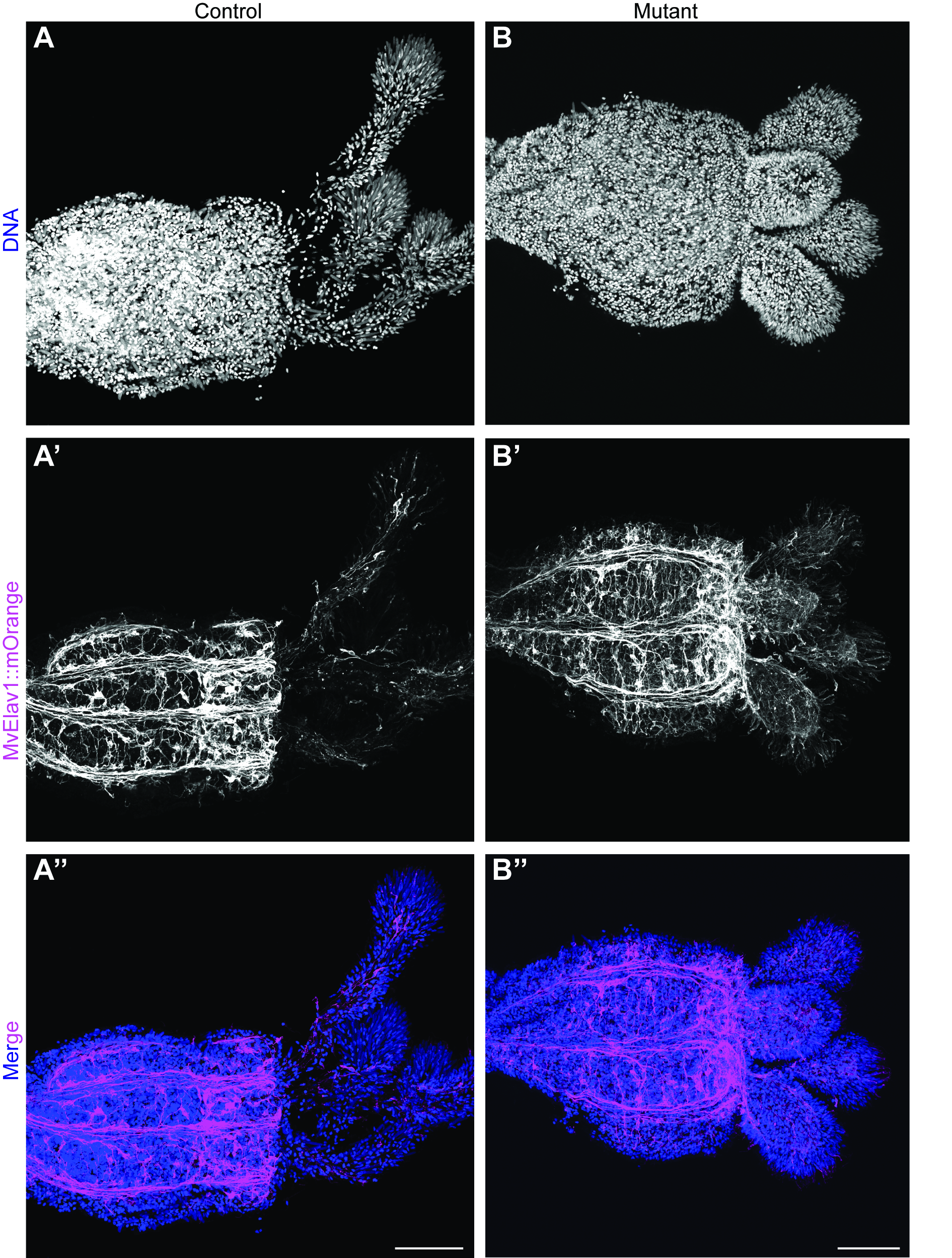

Supplement: Supplementary file 11 — Additional file 11: Fig S9. Loss of NvCoREST does not affect the NvElav1::mOrange+ nervous system. (A and B) Confocal images of immunofluorescence staining on control and mutant primary polyps showing DNA in blue and NvElav1::mOrange in magenta. Heterozygous NvCoREST mutant 1 animals were crossed to animals double heterozygous for NvCoREST mutant 1 and the NvElav1::mOrange transgene. The experiment was performed three times, independently and a minimum of 10 animals per genotype, per replicate were analyzed and showed the same result. Scale bars: 50 µm. [file 12915_2022_1385_MOESM11_ESM.tif]
